# Co‐designing interventions for chronic pain: A participatory action research study with south Asian women

**DOI:** 10.1111/bjhp.70072

**Published:** 2026-04-10

**Authors:** Sukhvinder Biring, Amy E. Burton, Peter Kevern

**Affiliations:** ^1^ Centre for Health and Development, School of Health, Education, Policing and Sciences Staffordshire University Stoke‐on‐Trent Staffordshire UK

**Keywords:** chronic pain, co‐designing interventions, participatory action research, South Asian women

## Abstract

**Objectives:**

Chronic pain is a significant health issue, particularly for women, with South Asian women being an underrepresented group in research. This study aimed to explore the needs and challenges of South Asian women living with chronic pain and develop strategies to address them.

**Design:**

Participatory Action Research.

**Methods:**

Sixteen South Asian women in the United Kingdom, aged 30 to 78 years, participated in three rounds of data collection through focus groups, co‐development of two intervention approaches and feedback sessions. Participants completed the full long‐form Warwick‐Edinburgh Mental Wellbeing Scale (WEMWBS) prior to phase 1 and again in phase 3. A paired‐samples *t*‐test was conducted using SPSS to determine whether there was a significant difference between pre‐ and post‐intervention scores.

**Results:**

Phase one identified two pain management intervention approaches: a tailored written resource and a group peer support intervention. Phase two provided insights into their design. Phase three evaluated the strategies following creation and pilot and identified key themes regarding effectiveness: resource design, effects and continued engagement. Quantitative analysis showed significant improvements in mental well‐being scores across the course of the Participatory Action Research process.

**Conclusions:**

This study highlights barriers and facilitators to pain management among South Asian women, offering transferable insights for culturally sensitive interventions. Participatory approaches can facilitate the development of culturally tailored interventions with the potential to enhance coping, self‐efficacy, empowerment and mental well‐being. This study provides methodological and practical guidance for co‐designing interventions for underrepresented communities, with implications for broader implementation and future research.

**Statement of Contribution**

**What is already known?**

- Women experience chronic pain more than men, yet their pain is often delegitimised, poorly managed or untreated.
- Social, cultural and psychological factors shape how chronic pain is experienced and managed.
- Literature on chronic pain among South Asian women remains extremely limited.

**What does this study add?**

- Offers insight into barriers and facilitators to pain management in South Asian women.
- Highlights cultural factors affecting pain experience and management in South Asian women.
- Provides a model for co‐designing and developing holistic interventions

## INTRODUCTION

Globally, approximately 1.5 billion individuals are living with chronic pain, which profoundly affects quality of life and wellbeing (Mills et al., 2019; Shetty et al., 2024; Yong et al., 2022) and chronic pain is a major public health problem that has high personal and societal costs (Breivik et al., 2006; Dueñas et al., 2016; Phillips, 2009; Yong et al., 2022). Ethnicity and sex (Bull et al., 2023; Goyal et al., 2015) are key factors influencing the experience of pain, with females being disproportionately affected (Fillingim et al., 2009; Koons et al., 2018; Mills et al., 2019).

Despite the profound impact, chronic pain for many women remains unresolved, underscoring the urgency of addressing women's needs to ensure equitable and effective care (International Association for the Study of Pain, 2021). Concerningly, those most affected by pain are often underrepresented in studies designed to address its management (Janevic et al., 2022) and knowledge about pain interventions has predominantly been shaped by studies involving participants who are ‘healthier, wealthier, younger and more likely to be White than the general population’ (Janevic et al., 2022). A recent systematic review has highlighted that understanding of South Asian Women's (SAW) experiences of chronic pain is particularly absent from the literature and in need of exploration (Biring et al., 2025).

This study aimed to engage SAW in an action research project to explore the needs and challenges faced when living with chronic pain and co‐develop and evaluate potential interventions. The research questions to be addressed were: (1) What are the key challenges faced by SAW living with chronic pain and what potential solutions could help address them? (2) How do SAW envision the design and content of interventions to help manage their pain?

(3) What are the experiences and perceptions of SAW regarding interventions they have co‐created? (4) Does participation in the action research process and associated intervention improve well‐being for SAW?

## METHODS

### Design

A participatory approach to action research was employed, which actively engages community members in research to create social change (McIntyre, 2008). Stringer and Aragón's (2021) model for action research was followed: look (identify problems/needs), think and act. Ethical approval for this study was obtained from the University of Staffordshire Health, Education, Policing and Sciences ethics committee (Reference: SU_22_076).

### Study sample

Sixteen women (Indian, *n* = 14, Pakistani, *n* = 2) between the ages of thirty and seventy‐eight years (*M* = 56.63, *SD* = 17.25) were recruited using purposive and snowball sampling. Demographic details can be found in Table 1. The shortest period of experiencing chronic pain was one year; the longest was fifteen years (*M* = 6.50). A range of sources of pain were reported: shoulder (*n* = 3), back (*n* = 4), knee (*n* = 3), abdominal (*n* = 2), neck (*n* = 1), neck and back (*n* = 1), neck and abdomen (*n* = 1), hands and wrists (*n* = 1).

**TABLE 1:** Demographics details of the participants.

ID	Age	Location	1st language	Length of UK residence (years)	Religion	Highest education	Marital status	Read/write English	Speak/understand English	Employment status	Pain location	Pain duration (years)	Pain level (1–10)
1	57	London	Punjabi	40	Sikh	High School	Married	Yes, basic level	Yes, basic level	Unemployed	Shoulder	5	6
2	46	West Mids	English	46	Non‐religious	Postgrad	Separated	Yes	Yes	Employed	Abdomen	1	6
3	67	London	Punjabi	51	Sikh	Primary School	Married	No	Yes, basic level	Retired	Hands/wrists	8	5
4	54	London	Punjabi	40	Sikh	High School	Married	Yes, basic level	Yes, basic level	Unemployed	Back	5	5
5	43	West Mids	Urdu	33	Muslim	High School	Married	Yes	Yes	Employed	Back	4	5
6	31	Berkshire	Hindi	25	Hindu	Graduate	Single	Yes	Yes	Employed	Neck/back	10	5
7	75	Berkshire	Punjabi	57	Sikh	High School	Widowed	Yes, basic level	Yes, basic level	Retired	Neck/abdomen	6	6
8	77	Berkshire	Punjabi	60	Sikh	Primary School	Widowed	Yes, basic level	Yes, basic level	Retired	Knee	15	5
9	44	London	Urdu	44	Muslim	High School	Married	Yes	Yes	Homemaker	Back	4	5
10	30	West Mids	Hindi	12	Hindu	High School	Married	Yes	Yes	Employed	Abdomen	5	5
11	78	London	Punjabi	62	Sikh	High School	Married	Yes, basic level	Yes, basic level	Retired	Knee	10	5
12	67	London	Punjabi	52	Sikh	High School	Widowed	Yes	Yes	Retired	Shoulder	8	4
13	57	London	Punjabi	42	Sikh	High School	Widowed	Yes, basic level	Yes, basic level	Unemployed	Back	3	6
14	32	London	English	32	Hindu	Graduate	Single	Yes	Yes	Employed	Knee	6	4
15	74	Berkshire	Punjabi	60	Sikh	High School	Married	Yes, basic level	Yes, basic level	Retired	Neck	6	5
16	74	Berkshire	Punjabi	65	Sikh	Primary School	Married	Yes, basic level	Yes, basic level	Retired	Shoulder	8	5

To track well‐being across the course of the study, participants completed the Warwick‐Edinburgh Mental Wellbeing Scale (WEMWBS) prior to phase 1. The WEMEBS has been extensively employed to assess the effectiveness of interventions for enhancing wellbeing (Blodgett et al., 2022). The total score can range from 14 to 70, with higher scores indicating higher levels of well‐being (Stewart‐Brown et al., 2009).

### Reflexivity

The first author, SB, is a female researcher who shares the participants' language and cultural background. This helped build rapport, foster trust and facilitate open and honest communication. SB does not have experience with chronic pain; this was shared with participants and they were reminded of their position as experts of their own experiences.

## PHASE ONE

Phase one represented the ‘Look’ part of the process (Stringer & Aragón, 2021) and sought to address the research question: (1) What are the key challenges faced by SAW living with chronic pain and what potential solutions could help address them?

### Procedure

Focus groups were chosen to generate rich data and allow participants to discuss problems and offer potential solutions (Onwuegbuzie, 2018). Three focus groups were conducted in December 2023 on days, times and locations that were convenient for participants. Two were conducted with six participants and one with four in line with guidelines on ideal group size (Kitzinger, 1995). Two were conducted in person (in the homes of two participants) and one online; discussions were held in English and Punjabi.

The facilitator, SB, used a prompt sheet of questions to guide the discussions in alignment with the GROW model (Whitmore, 2001), which offers a clear and structured pathway for goal setting, assessing current reality, exploring a spectrum of options and committing to a plan of action (see Table 2). At the end of each focus group, key points were summarised to allow participants to reflect, verify, clarify or add further contributions. Each group lasted approximately one hour and was followed by a debrief including contact information for support services.

**TABLE 2:** Focus group question guide for each phase.

Phase	Focus group schedule
1	*Implementing a coaching approach (G (goals) R (reality) O (options) W (way forward/will do))* What issues would you like to discuss and/or improve? (goal) What do you find most difficult about living with chronic pain and what areas would you like to change? (goal) Which would you like to focus on, and which are most important to you? (goal) How would you like things to be? (goal) What are the major challenges that you find stopping you from living well with chronic pain? I also requested if you could note any moments that you did not experience pain in the week or so prior to this discussion, if we could also discuss what you were doing and how you were feeling during or prior to this time. Do any of you wish to share your notes relating to these moments during the last week and what you were doing and how you were feeling? When are you not so aware of pain? Was your pain particularly bad on any day during the week and what were you doing and how were you feeling? When do you most notice your pain? What have you found that helps you or makes your pain better? (options) What is working well at the moment? (options) What have you found to improve things? (options) What do you think could improve things for you? (options) Reality: On a scale of 1–10 what number are you at in terms of your knowledge of chronic pain as in the reasons of why it can occur and the factors that can make it worse or better? One being not good at all and ten being excellent. You can write these down and I can collect it at the end if you prefer On a scale of 1–10 how confident do you feel at the moment in terms of managing the low mood or feelings of sadness, stated in the interviews? What number would you like to be at? (goal) What would you like to know and understand? How should be go about addressing these and what are the objectives? What would you like? What should we do? What ideas do you have? (Moving forward) What would be the best way? What do you think should be our first step? Is there anything else that anyone would like to add or talk about that we have not discussed?
2	*Resource* Let us discuss a specific example of a resource provided that you've brought to the group today: What aspects do you appreciate about it, and is there anything you dislike or would change? Do you like how it is presented? How would you prefer it to be presented? Do you like the pictures? Would you like pictures to be included? Do you like the colours? What colours do you like? What about the size of the booklet? Is there anything about how it is presented that you do not like or feel could be improved? Reflecting on your own experiences, what tips or advice would you offer to others who may be dealing with similar challenges in pain management? Also, what would you like to include that has been most helpful to you? What do you hope to gain from this resource for yourself? How do you hope for it to help you? Is there anything else you would like to add or discuss? *Women's circle* COM‐ B framed questions: Capability: What activities would you like to include? Do you all feel comfortable with engaging in these activities? Do you all feel confident in your ability to participate in the planned activities? Opportunity: Do you have access to the necessary materials and space to engage in these activities? (resources and environment) Do you need help organising these meetings/circles? Is there anything I can assist you with in organising or setting up the women's circles? Are there any particular days or times that work best for you to attend these gatherings? Can you all make this time, and will it be okay with your family circumstances and commitments? Motivation: Would you be interested in continuing these meetings and incorporating some of the activities that you all enjoy? Are there any personal goals or aspirations you would like to pursue within the context of the women's circle?
3	*Pain management booklet evaluation* Having had a chance to view the booklet and read through it, I wanted to discuss your thoughts and opinions about it. What did you think about the presentation, information and the pictures? What are your opinions after reading it? Do you feel that we have created what we intended? Does it meet your expectations? We discussed during our first meeting that you felt your knowledge about pain and the factors that can influence it was not very good, and you did not feel very confident in managing low moods. Has this changed now? If so, in what ways? What aspects (if any) did you like and/or which ones (if any) did you find unhelpful or dislike? Are there any things that are lacking in the resource and that can be improved or included? Are there any other changes that you would like to see? Is there anything else that you would like to share about the resource? *Women's circle evaluation* Can we discuss your experience of participating in the weekly women's circles? How did you find the experience? What were your main feelings and emotions experienced when participating in the circles and generally in life now? Have you noticed any changes in your daily life, health, and wellbeing? If so, can you please provide examples of any changes? How content would you say you are with life in general now? How likely are you to continue participating in these circles? Would you recommend these circles for managing pain and wellbeing? Is there anything else that you would like to share?

### Data analysis

Transcription was completed by the first author. Focus groups conducted in English were transcribed verbatim. Focus groups conducted almost exclusively in Punjabi (*n* = 2) were contextually translated to preserve participants' intended meanings, as direct word‐for‐word translation between Punjabi and English can distort meaning (Barbour, 2018; Esposito, 2001).

Transcribed data were analysed using Reflexive Thematic Analysis (Braun & Clarke, 2021), guided by a critical realist framework, encompassing ontological realism, epistemological constructivism and an inductive approach to ensure the findings remained close to participants' reported experiences without imposing predetermined assumptions (Kvale, 2007; Maxwell, 2012; Ormston et al., 2014).

Aspects deemed potentially relevant to the phase's objectives were identified and labelled with meaningful codes. Similar codes were combined, and from these initial codes, themes were generated to reveal shared patterns across the dataset. Thematic maps offered a structured visual representation of interconnected themes in phase three. A reflexive approach was supported by reflexive journaling (Braun & Clarke, 2021; Dodgson, 2019) alongside discussion of developing themes with the second and third authors. Emerging codes and preliminary themes were discussed with the second and third authors, who contributed to refining the thematic structure by critically questioning interpretations and agreeing on the final themes to be retained. Participants were contacted by phone in December 2023 to review and provide feedback on the synthesised findings as a form of member checking (Braun & Clarke, 2021).

### Results

Three themes were developed: (1) Holding Space: Sharing, Safety and Solace; (2) Understand My Pain: Self and Society; and (3) Restoring the Losses and Creating Positive Experiences.

#### Theme 1: Holding space: Sharing, safety, and solace

The group described the need for space to be held for them and highlighted the therapeutic benefit gained by taking part in candid conversations concerning their experiences of pain in a safe, non‐judgmental setting. Many expressed relief in the compassionate space provided, and this freedom from judgement appeared to offer solace, lifting a burden they often carried in silence:

I found the interview [speaking about another study they had been part of] so helpful to me, like a heavy burden was lifted from my shoulders. I felt lighter [laughs] but today [being part of this study] it is like multiple burdens have been lifted. (FG1)

The accounts contrasted this feeling of safety with other settings where they felt they were regarded as complaining:

Everyone here understands what pain feels like; we can talk openly, I can't at home as it would be seen as me complaining […] no one listens and makes you feel like you are overexaggerating. (FG3)

This lack of support in other environments emphasised the importance of finding a supportive community to be part of. Many participants were empathetic and offered validations to others, highlighting how the shared experience of the group provided understanding and recognition. One participant shared how she wished that health care providers (HCPs) could emulate the empathetic atmosphere of the focus group:

I wish doctors and other people could listen like this. I know the number of times I left my surgery in tears. (FG1)

This poignant admission also highlights a seemingly unmet need for compassionate and attentive listening from those in healthcare settings. It appeared that their high regard for sincere compassion stemmed from the rarity with which they encountered it, both in their interactions with HCPs and in their encounters with others. Summarised by another participant who stated:

This is the first time in my life that anyone has actually listened and understood my pain. (FG2)

#### Theme 2: ‘Understand My Pain’: Self and society

When asked what factors were most important to address and which would help them, the women highlighted the significance of understanding their own pain and a desire to learn strategies to manage it, in addition to a need for others to understand their experience. There was, therefore, a dual desire for self‐understanding and a societal understanding of pain:

If you are asking which are most important then for me, it [is] to understand my pain and not only me but other people too. (FG1)

The women expressed a need for others to understand the depth of their pain and the challenges associated with living with it:

I need others to understand what our pain is and how difficult it makes life, and it is not just us complaining about nothing…I need to understand what I can do to help make things easier for me. (FG3)

There was a shared belief that by shedding light on the participants' struggles, they could cultivate empathy and compassion in others, establishing a pathway for support:

People can help when they understand the burden of this pain. (FG2)

There was also a shared desire for something that may assist in managing pain while enabling them to articulate their pain experience to others. The participants proposed suggestions for written resources that could help others to understand:

I think if people can see it written down…then people believe it more. I think it strengthens our experiences and difficulties. (FG3)

I have seen some books at the hospital…I think something like that for us but what we can read. (FG1)

The participants agreed that something ‘*written down…in a leaflet or booklet type format*’ would be particularly helpful.

#### Theme 3: Restoring the losses and creating positive experiences

Participants also expressed a deep sense of loss, missing aspects of their lives such as employment and enjoyable activities, many of which had ceased following the onset of pain. Reflecting on the losses, the accounts suggested these had resulted in deteriorating mood and increased pain:

I miss talking with other colleagues during tea breaks, you got to laugh a bit in the day even with pain and that helped my mood. (FG1)

I miss the joy in life that pain takes…I would not say I am in a good place. (FG3)

The discussions also emphasised how household duties and pain consumed the participants' time, leading to the neglect of activities for themselves and moments that brought joy:

I miss the good things pain has taken, and I feel what is left is just housework which never seems to finish or then pain. (FG1)

Many accounts highlighted how chronic pain had become a significant barrier, impacting their ability to engage in enjoyable activities and affecting their overall quality of life. Examples reflected a common sentiment that SAW were expected to prioritise the needs of others over their own:

I do not know the last time I did anything for me, for my happiness. (FG1)

I think in our community, the women make time for everyone else but themselves. (FG2)

I am missing things that make life fun…everything feels like a chore. (FG3)

The last quote captures the broader impact of chronic pain. Instead of living a full and enjoyable life, participants described feeling trapped in a cycle of pain and daily chores, which contributed to a sense of existence rather than active living.

The participants also unanimously expressed the power of joy and happiness in mitigating their pain. Pain‐free moments were associated with activities that elicited positive feelings, emphasising the reciprocal relationship between mood and pain perception:

When I am doing something I enjoy, I forget the pain. (FG1)

As the conversations evolved, the potential of embracing activities that participants had previously enjoyed, fostering connections with others and actively engaging in and replacing the losses they had endured became apparent:

I enjoyed my walk today. I have usually not had reasons to go out. I should walk more. (FG1)

I miss talking with other colleagues…so this has been very nice. (FG1)

Upon the request for the participants to reflect on past activities that brought them joy, they recollected numerous experiences, including crochet, knitting, reading, art, music and painting. It was clear that there was a potential avenue towards improving the participants' well‐being by enriching their lives with joyful moments, which could help diminish their experiences of pain or, at the very least, provide more pain‐free moments as well as a distraction.

## PHASE TWO

This phase represented the ‘Think’ part of the process (Stringer & Aragón, 2021) and addressed the research question: How do SAW envision the design and content of interventions to help manage their pain?

### Procedure

A second round of focus groups took place in February 2024 to discuss the findings of phase one. The composition of the groups remained unchanged, and the sessions were organised and conducted in the same manner. The groups lasted approximately forty‐five minutes and focused on the design and content of the intervention strategies proposed.

Firstly, in phase one, the groups all expressed the desire for opportunities that enabled them to meet and connect with others and recommence enjoyable activities. The first author proposed a women's circle to meet this need as it aligns well with both Carl Rogers' (Rogers, 1946) person‐centred approach and a biopsychosocial‐spiritual model (Hasenfratz et al., 2021; Sulmasy, 2002). After confirming that a women's circle would be acceptable, participants used Phase Two focus groups to identify what the circles should include and how they should be structured.

Secondly, participants explicitly expressed their desire for a written resource. To explore this further, the first author asked the women to look at resources they were already aware of and bring these to the focus groups to discuss. The discussion considered these resources and explored requirements for the content, layout and design of the new written resource.

To inform the design of the women's circle, discussion questions were framed using the COM‐B model (Michie et al., 2011) to explore participants' engagement in activities aimed at enhancing wellbeing and supporting pain management, ensuring that the activities would be appropriate and engaging for all participants. Insights from these discussions were used as a ‘behavioural diagnosis’ to guide intervention content. Activities were selected to match participants' abilities and preferences; for example, some could not knit, while others wanted to include it. This ensured that the women's circles were feasible, inclusive and engaging for all participants. Barriers were addressed as they arose: if capability was a limitation, activities were chosen that could be achieved; if interest was low, alternative activities were selected; and if resources were an issue, arrangements to provide such resources were made in advance.

The questions explored access to the necessary resources and supported identification of activities that would be suitable and enjoyable for everyone involved (see Tables 2 and 3).

**TABLE 3:** The participatory‐action research co‐design process.

Stage	Guiding models/frameworks	Primary goal of focus groups/feedback sessions	Key aims/outcomes	Participants' voices/needs
LOOK	GROW	Identify challenges, beliefs, attitudes, barriers and goals	Deep understanding of lived experience, psychosocial and spiritual needs, beliefs, attitudes, social norms and barriers to more effective self‐management	Countless losses, low mood, feeling misunderstood by health professionals Lack of resources Desire for knowledge on chronic pain, culturally sensitive resources/interventions, meaningful social connection and emotional support Importance of religion and spirituality Pain‐free moments occurred when engaged in activities they enjoyed
THINK	GROW, COM‐B	Co‐design culturally tailored and behaviourally informed interventions	Co‐created women's circles and written resources; interventions aligned with participants' motivations, capabilities, social context, and values; incorporated spirituality and activities that bring joy, meaning and purpose	Desire for connection, biopsychosocial‐spiritual approach and activities that bring joy and reduce pain Participants shared their ideas for a resource (the structure/layout) and selected the colour scheme
ACT	GROW, COM‐B	Implement, evaluate, and adapt interventions based on participant feedback	Enhanced well‐being, empowerment, self‐efficacy, pain management, and engagement; strengthened sense of meaning and purpose; interventions iteratively refined to reflect participants' needs	Ownership of sessions Sharing stories, affirmations, faith‐based practices Validation, knowledge, empowerment, and meaning Engagement in enjoyable and meaningful activities

### Data analysis

Data were transcribed and analysed using Reflexive Thematic Analysis as described for phase one.

### Results

#### Women's circle

In alignment with duration recommendations for self‐management interventions for chronic conditions (Lorig et al., 2006), a six‐week intervention was planned. As the women had reported low moods, SB contributed to the intervention development by providing advice to the group regarding evidence‐based strategies to enhance mental well‐being. These were informed by the NHS’ ‘five steps to mental wellbeing’ recommendations, including connecting with others, encouraging physical activity, learning new skills, giving to others and mindfulness (NHS, 2024). Activity decisions were made collaboratively to include a diverse range of activities, including origami, painting and knitting. Each of the groups decided upon their own schedule of activities (see Supporting Information for weekly sessions). It was agreed that the groups were to be for two hours each week and be led by the participants, but also facilitated and coordinated by SB. The circles began in February 2024 and were evaluated after six weeks.

#### Written resource

The focus group discussion in phase 2 highlighted three key areas of requirements for the written resource: (1) Design: Layout and Visual Elements; (2) Content; and (3) Intended Impact.

##### Layout and Visual Elements

Participants wanted the resource to be presented in light colours and pastel shades, with pink being particularly favoured. They described these colour choices as calming, uplifting and soothing.

I really like pink; it is a very calming colour that can lift your mood too. (FG1)

I find pink quite soothing. (FG3)

They felt a combination of images and text presented was ‘a good idea’, preferred a balanced text‐to‐image ratio (‘half and half’), and wanted something they could ‘flick through’ that looked ‘nice, simple, colourful and not depressing’. They specifically mentioned a preference for images that complemented or illustrated the written text, as they found this format easier to understand and engage with:

when we were at school it was pictures that helped us. (FG2)

I can't really just look at just text, I need images. (FG3)

##### Content

The participants highlighted the significance of raising awareness about the invisibility and impact of pain and its influencing factors and wanted to increase their knowledge of the latter and others' understanding of the impact of chronic pain. They wanted the ‘burden of pain’ to be understood, and to emphasise that the absence of visible damage on imaging tests did not discount the presence of pain

People need to understand that just because nothing comes up on the scan does not mean we are lying. (FG2)

They will only have compassion if they understand how much it affects someone. (FG2)

They also reported that they did not feel confident about their knowledge of factors that could influence pain and wanted the resource to provide information on this:

I do not think we know things we might be doing to make our pain worse. (FG2)

Agree and any other things that can make it better. (FG2)

Participants went on to contribute their personal tips and experiences, including the benefits of pets, sleep support and massage devices and the importance of prayer, mindfulness techniques and relaxation exercises. Each participant shared something that helped improve their pain or well‐being to be included in the resource.

##### Intended Impact

Raising awareness and understanding among others in their broader social circles was highlighted. There was a shared desire to take control of their wellbeing, be in a ‘better place mentally’, and find ‘some peace’, ‘calmness’, contentment and freedom from ‘negative emotions’ amidst the challenges of managing their condition. They wanted their shared tips combined so that they could refer to them daily and were striving for ‘more good days than bad’:

I just want to know what else others are doing and that I can to do myself to be comfortable and content and get me to a better place mentally too and things I might be doing that is making it [pain] worse. (FG3)

I agree on the better place mentally and I just want to add a place of comfort without all the negative emotions…with all the things we are learning from each other to be summarised in one place. (FG3)

Participants envisioned the resource as a tool for empowering them to manage their pain and well‐being independently.

## PHASE THREE

This phase represented the ‘Act’ part of the process (Stringer & Aragón, 2021) and addressed the research questions: (1) What are the experiences and perceptions of SAW regarding interventions they have co‐created? and (2) Does participation in the action research process and associated intervention improve well‐being for SAW?

### Procedure

Following phase two, the self‐management written resource (see Supporting Information) was produced by the first author based on the recommendations of the participants. The first author combined the contributions from participants using insights from other studies on the production of written educational resources (Adepu & Swamy, 2012; Griffin et al., 2003; Hoffmann & Worrall, 2004; Leake et al., 2021) and the DISCERN tool, which supports assessment of the quality of written health information (DISCERN, 2024). The latter was applied to inform and evaluate the design of the self‐management resource, checking that each criterion was addressed. The women's circle interventions were also commenced and facilitated.

Phase three evaluated these intervention strategies using both quantitative and qualitative methods. This approach reflects a convergent design, wherein both qualitative and quantitative data were collected and combined during the analysis and interpretation stage (Fetters et al., 2013).

Three focus groups were conducted in March and April 2024 (see Table 2 for the prompt sheet). The same steps for conducting the focus groups were followed; however, all focus groups in this phase were conducted in person. In addition, participants completed a second WEMWBS questionnaire.

### Qualitative analysis

Data were analysed using Reflexive Thematic Analysis as described in phases one and two. Member checking of the findings was conducted in person with each group during their subsequent meetings.

### Quantitative analysis

#### Data diagnostics

The difference between pre‐ and post‐WEMWBS scores for each participant was calculated. Examination of the distribution of these differences through Q‐Q plots and histograms indicated a normal distribution, which was further confirmed by both the Kolmogorov–Smirnov and Shapiro–Wilk tests of normality.

#### Analytic strategy

A paired‐samples *t‐*test was conducted to explore whether there had been a significant improvement in the WEMWBS scores over the course of the study.

### Results

#### Quantitative

There was an increase in participants' mean WEMWBS post‐intervention scores from the baseline values, indicating an improvement in wellbeing. Pre‐intervention WEMWBS scores (*M* = 38.31, *SD* = 3.55) increased on average by approximately ten points (95% CI [7.61, 12.76]) at the six‐week mark (*M* = 48.50, *SD* = 3.93). This increase was significant (*t* (15) = 8.43, *p* < .001, two‐tailed; *d* = 2.11), indicating a large effect (Cohen, 1992; Lakens, 2013).

#### Qualitative

**FIGURE 1:**
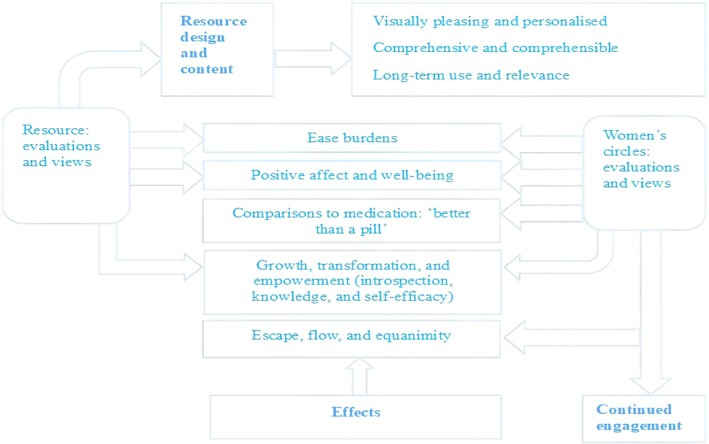
Thematic map.

Three key themes were developed: (1) resource design and content, (2) effects and (3) continued engagement (see Figure 1 for a thematic map).

### Theme 1: Resource design and content

All expressed overwhelming satisfaction with the aesthetics of the resource, appreciating that it was both visually pleasing and personalised. Descriptions of the aesthetics included: ‘pretty’, ‘nice to look at’ and ‘stunning’. From the perspective of being personalised, they felt it was effectively tailored to their needs and viewed it as a ‘great little guide’ to managing their pain and wellbeing. They felt it contained information that addressed their concerns and incorporated strategies of interest and relevance to them:

I loved it and thought it was so beautiful. (FG3)

I love that it is everything we said we wanted, needed, and so much nicer than I thought it would be. (FG3)

The women also valued the resource's clarity, finding it easy to understand. They appreciated that everything they needed was summarised in one place, making it a comprehensive yet accessible guide to managing their pain and wellbeing:

The way it is written is great, I mean you can understand everything, and it is actually nice to read. (FG3)

Furthermore, they noted that the resource effectively conveyed the impact of pain, allowing others to understand their experiences:

It presents an accurate picture of what it is like living with pain…

I think people reading it would understand the difficulties we face. (FG3)

Participants also felt the strategies could be implemented by people of all ages and throughout their lives. Overall, it was viewed as a valuable tool that could support their health and well‐being over the long term:

[it] addressed things that apply to all ages, and I know everything in here would be useful to me now as it would ten or twenty years from now. (FG3)

### Theme 2: Effects

Participants described diverse changes and experiences from engaging in the project. Some findings regarding effects were applicable to both the resource and the women's circle; others were only relevant to one intervention strategy.

Combined impacts of both the women's circles and the resource included the feeling of burdens being eased and a sense of relief or liberation. One participant shared a particularly moving reflection:

My world had started becoming really heavy for me, the pain of feeling like you are not important, mad, no one wants to listen…cares or understands. I just wanted someone's understanding…I feel a lot of weight has been lifted from me through speaking with everyone [in the women's circles] and this [written resource] did that too. (FG1)

The act of voicing and having experiences acknowledged and reaffirmed in the circles and visually reflected in the resource appeared to provide the participants with a sense of validation, providing comfort amid external skepticism and lack of understanding. There was also agreement that the resource would ease the burden of explaining their condition to others, which held promise for emotional benefits:

I do feel this will help so much in taking the pressure of explaining my situation… before I would try only for no one to still get it. (FG3)

Participants also reported gaining insights into various aspects of self‐care and pain management strategies from other participants through both the circles and the written resource. There was evidence of transformations, such as significant changes in their understanding of pain and their abilities to manage it. There was an indication of an increased awareness regarding the holistic approach necessary for managing their pain and well‐being:

I have become much more knowledgeable on pain and mental health which I really did not know were connected. (FG3)

Specifically, the women's circles were seen as spaces facilitating genuine acts of kindness and compassion. Participants expressed leaving the circles feeling ‘energised’, ‘lighter’, ‘recharged’ and ‘relaxed’:

I always leave feeling happy, relaxed, and just more lighter. (FG1)

you just feel more positive, energised. (FG3)

You tend to forget about your problems and worries. (FG3)

Furthermore, for many who had given up work due to pain, the circles filled a void left by this loss of routine, providing space for social interactions and a sense of purpose. They appreciated the opportunity to reconnect with others and engage in meaningful activities that they enjoyed. As one participant reflected,

I now have a better routine as I feel better in myself and when you feel better you can do more things…I am doing things that I like and make me happy, and I get to speak with nice people. I did not have this in my life. (FG1)

Participants also valued the escape from household chores and other obligations that the circles provided, describing a loss of awareness of time, feeling a ‘sense of peace’ and being less impacted by ‘annoying stuff’. They spoke of how the circles provided a ‘break’ from usual chores, a ‘change’ from tasks that did not really fulfil them or bring them a sense of purpose:

I really look forward to these group meetings, it makes a change from boring housework. (FG1)

Participants also expressed a sense of calm, a ‘sense of peace’, and better able to manage their emotions and stress. They felt that they were less impacted by negative emotions:

You find yourself not stressing about petty things so much as you have better things going on and to look forward to. (FG3)

They also expressed optimism about the future and felt that they were ‘climbing higher up out of the dark place’ they had initially reported finding themselves in:

I am living a life I was not living a few months ago. (FG1)

Now I make time for things that make me happy and matter to me. I've learned the importance of self‐care and prioritising things that make me happy for helping with my pain. (FG3)

### Theme 3: Continued engagement

Participants described the benefits of engagement in the women's circle and the value they placed on the learning provided by the written resource. They highlighted that they viewed these benefits as long‐lasting, with the potential to continue to be of benefit long into the future.

The benefits were compared to their previous methods of coping; for example, the women's circle was felt to be more effective than taking medication for pain. They spoke of how the positive moods and emotions appeared to last long after the circles ended:

This is better than a pill, as the improvements last longer. With a pill, you really are back to feeling exactly the same in no time, and the other changes we just spoke about, that pills can't give you. (FG3)

This has given me things that no medicine could, some happiness, peace of mind, a long break from pain. (FG2)

Motivation came from the profound changes experienced since participating in the project and engaging with the interventions. One participant summed this up by describing a transformation in which they were no longer being consumed by pain:

All I used to do was think about all the work I had to do and pain; this is not what my life is like now. I feel the quality of my life is much better and it is not all about pain and sadness. (FG1)

Participants were committed to remaining actively involved in the women's circles and managing their pain and wellbeing. All expressed a desire to continue attending the circles and engaging in similar activities in the future, further demonstrating their commitment to sustaining the positive outcomes derived from their participation:

I will definitely keep continuing with them and making more changes. (FG3)

These small changes are making big improvements in my life…and I hope we all can continue meeting like this. (FG1)

## DISCUSSION

This study used a participatory action research approach, which integrated the GROW (Whitmore, 2001) and COM‐B model (Michie et al., 2011) to guide participant‐led exploration, goal setting and problem‐solving to co‐create and develop intervention strategies to help SAW living with chronic pain. The first phase explored the key challenges that SAW with chronic pain face as well as potential solutions. The second phase used this data to co‐create two intervention strategies, and the third phase evaluated these intervention strategies and overall participation in the project.

The study identified several key barriers to effective pain management for SAW, including limited knowledge about chronic pain, understanding the interaction between mental and physical health, prioritising others over self, the demands of extended families, negative encounters with HCPs, limited confidence in engaging in self‐management activities, and lack of access to suitable resources. Researchers included culturally tailored group activities, peer social support and participatory co‐creation of interventions. To enhance engagement and effectiveness, intervention developers, practitioners and policy makers should provide accessible, culturally sensitive resources, support group‐based activities and actively involve participants in planning and tailoring interventions.

Both the qualitative and quantitative evaluations indicated that participants experienced improvements in well‐being. The mean WEMWBS post‐intervention scores increased from the baseline values with a significant improvement of approximately ten points representing a clinically meaningful change. The categorical interpretations provided by the Warwick Medical School (2023) provide additional context. Most pre‐intervention scores (81.25%) fell within the low well‐being category (scores ≤ 42) and three out of sixteen participants (18.75%) scored within the range of moderate well‐being (scores greater than 42 but lower than 60). By the end of the intervention, all scores had increased, falling in the moderate well‐being range, which is a promising outcome.

Triangulation of this finding with the qualitative data supports a conclusion that these improvements in wellbeing appeared to be linked to engagement in the research process. The responses were overwhelmingly positive, with many describing a transition from a state of darkness to one of light. They described the written resource as empowering, supporting self‐education, facilitating the sharing of their experiences, and enhancing understanding among others. In addition, the women's circles were celebrated as a source of support, connection and opportunity to re‐engage with enjoyable activities. Overall, the experience was presented as empowering and providing a sense of purpose and joy.

An integral part of this project was the use of focus groups, which provided a forum for SAW to share their experiences in a supportive environment. The focus groups served a dual purpose: collecting qualitative data and providing a supportive group environment that promoted social connection, empowerment and shared learning. This suggests that practitioners may also benefit from facilitating similar group sessions, where participants feel heard and supported in addition to contributing to research insights.

By co‐creating the intervention, the project ensured that the solutions were directly relevant to SAWs' needs and contexts. In this project, the GROW model (Whitmore, 2001) provided an effective framework for exploring SAW's goals and needs (Table 2). It acknowledged participants' unique perspectives and expertise, validated their experiences of pain and facilitated active listening and learning from them.

Engaging in active listening, within a focus group format, can be viewed as an act of ‘rehumanising’, recognising and valuing participants' experiences, emotions and perspectives (Rogers, 1951). While empathy and compassion lay the groundwork, they alone are not sufficient for facilitating change. Accountability, setting goals and motivation are also critical components for facilitating change (Deci & Ryan, 2014; Michie et al., 2011; Whitmore, 2001). This project, therefore, also integrated behaviour change theory grounded in the COM‐B model (Michie et al., 2011) to understand what motivates SAW with chronic pain and involve them in goal setting, intervention planning and decision‐making processes. This approach can help foster a sense of ownership and accountability, increasing the likelihood of sustained participation and commitment (Maini et al., 2020; Starr, 2021; Whitmore, 2001), which was illustrated in the participants' accounts of intentions to use the written resource regularly and continue the women's circles beyond the project's end.

Previous literature has emphasised the value of social support and group‐based interventions for helping individuals better manage pain and reduce pain severity and negative mood in chronic pain patients (Allen et al., 2015; Franqueiro et al., 2023; Jensen et al., 2011; Wilson et al., 2022). Historically, these studies have included limited detailed data on ethnicity, with some reporting only the percentage of White participants or omitting such information entirely (Franqueiro et al., 2023), meaning that the transferability of these findings to SAW has not been well understood (Biring et al., 2025). South Asian populations are underrepresented in research, with language, cultural barriers, and mistrust of research known to be common barriers to engagement (Quay et al., 2017). The present study has illustrated that such populations can successfully be engaged in research and that a culturally sensitive, collaborative and participant‐led approach is an effective method for engagement. Additionally, such an approach can create culturally acceptable and effective intervention strategies for SAW, which can lead to measurable improvements in well‐being for this population.

Patient‐centred care, characterised by appropriateness, emotional and physical support and delivery with respect for patients' needs, beliefs and preferences, is known to enhance patient satisfaction and improve chronic disease self‐management (Gordon et al., 2017; Rathert et al., 2013; Vakil et al., 2023; Wagner et al., 2001). The present study provides a practical example of this with SAW. Participants were given choice and control over the development of the interventions in line with their preferences. For the women's circle, they chose an activity‐based group which aligns well with evidence that participating in crafts and enjoyable activities can be beneficial for mental well‐being (Blodgett et al., 2022; Bloem et al., 2018; Curry & Kasser, 2005; Mak et al., 2023). This strategy was therefore both patient‐centred and evidence‐based. As art and craft‐based interventions represent practical and cost‐effective strategies for managing pain and enhancing wellbeing (Crafts Council, 2020), these approaches hold promise for the development of future interventions for this population.

### Limitations

While there is potential for participants to provide socially desirable responses to evaluation questions (Acocella, 2012), efforts were made to mitigate this. The establishment of rapport and mutual respect within the group sought to foster an environment conducive to openness and honesty, where participants felt at ease expressing their views candidly. Furthermore, participants were prompted to engage in discussions regarding both favourable and unfavourable elements of the intervention and resource. Additionally, the intervention was designed through collaborative efforts, where participants were active contributors. By doing so, participants were empowered to provide genuine feedback, including any aspects they were dissatisfied with, to ensure the interventions met their needs.

## CONCLUSION

The successful co‐creation of a resource that participants valued, along with the establishment and planning of women's circles that were also found to be beneficial, demonstrates the importance of participatory approaches. This collaborative approach not only empowered participants but also ensured that the intervention and resources were tailored to their needs and preferences, leading to engagement and self‐reported satisfaction.

The insights gained provide valuable direction for developing new interventions for managing chronic pain. This study highlights the transformative impact of engaging in participatory research, which recognises and addresses the unique experiences of underrepresented groups. Participatory approaches should be prioritised to ensure that the perspectives of those for whom interventions are designed are fully reflected. Co‐created resources and group‐based activities fostered autonomy, choice and social support, contributing to meaningful improvements in wellbeing in this study. These findings underscore the importance of moving from researcher‐led to participant‐led, co‐developed approaches that are responsive to participants' needs, preferences and lived experiences. Empowering individuals through research and tailored interventions can not only enhance their wellbeing but also contribute to the broader effort of fostering more equitable and effective healthcare practices.

## AUTHOR CONTRIBUTIONS

**Sukhvinder Biring:** Conceptualization; methodology; data curation; investigation; formal analysis; project administration; writing – original draft; writing – review and editing. **Amy E. Burton:** Conceptualization; methodology; formal analysis; supervision; writing – review and editing. **Peter Kevern:** Conceptualization; methodology; formal analysis; supervision; writing – review and editing.

## CONFLICT OF INTEREST STATEMENT

The authors declare no conflict of interest.

## ETHICS STATEMENT

The study was approved by the Ethics Committee at Staffordshire University (approval number: SU2257). All participants provided written informed consent prior to enrolment in the study.

## Supporting information


Supplementary Material 1:

Supplementary Material 2:

## Data Availability

Data is available from the corresponding author by request.
