# Supplementary material for: Co‐designing interventions for chronic pain: A participatory action research study with south Asian women
Source: Br J Health Psychol. 2026 Apr 10;31(2):e70072. doi: 10.1111/bjhp.70072 (PMC13067331; doi:10.1111/bjhp.70072)
Supplement: Supplementary file 1 — Supplementary Material 1: [file BJHP-31-0-s002.docx]

**Supplementary Material**

Women’s Circle Weekly Activities

|  | Group 1 | Group 2 | Group 3 |
| --- | --- | --- | --- |
| Week 1 | Tea, origami, playing cards | Tea, Book club, talking | Tea, cards, draughts, prayers |
| Week 2 | Cooking a | Cooking | Cooking |
| Week 3 | Art: colouring | Painting | Art: colouring |
| Week 4 | Knitting/ crochet | Diamond art | Knitting/ crochet |
| Week 5 | Continue knitting/crochet | Continue diamond art | Continue knitting/crochet |
| Week 6 | Reflection/repeated activities | Reflection/repeated activities | Reflection/repeated activities |
