# Supplementary material for: Co‐designing interventions for chronic pain: A participatory action research study with south Asian women
Source: Br J Health Psychol. 2026 Apr 10;31(2):e70072. doi: 10.1111/bjhp.70072 (PMC13067331; doi:10.1111/bjhp.70072)
Supplement: Supplementary file 2 — Supplementary Material 2: [file BJHP-31-0-s001.pdf]

Chronic Pain in Women

Living Well with Chronic Pain

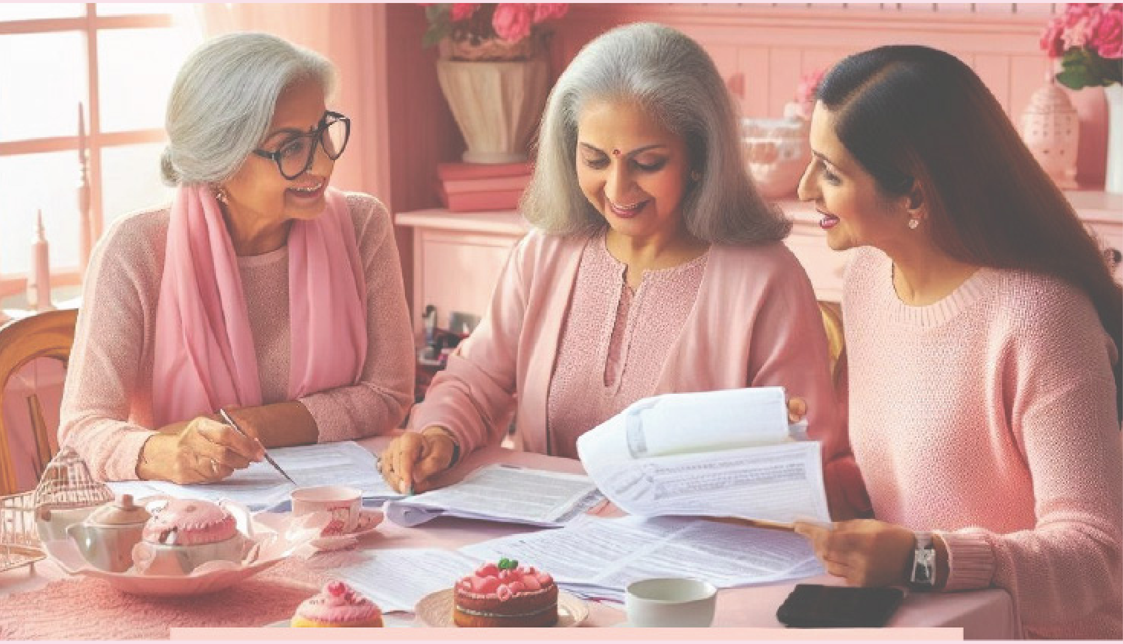

Information for patients, relatives, and friends

Introduction

This booklet provides information about chronic pain, challenges experienced by patients living with it, factors that can affect an individual's level of pain, and strategies for managing it. This is not meant to replace consultation between patients and their health care providers. However, it aims to help enhance understanding and well-being and help patients manage their pain with non-medication-based alternatives. Exploring and applying different self-management strategies is key to enhancing overall well-being. Without proper management, pain can lead to a decline in health and mental well-being. Every individual's situation is unique, so finding personalised solutions that work for each individual is key.

What is pain?

Pain serves as the body's warning system, signalling that something might be wrong or requires attention. Acute (short-term) pain functions like a swift alarm, triggered when we get injured or may get hurt. Special nerves send a message to the brain, prompting us to take immediate action, such as removing our hand from a hot object or resting a sprained ankle. Typically, this type of pain diminishes as the body heals. However, in some cases, even after the healing process, the brain may persist in signalling pain, leading to chronic pain.

What exactly is chronic pain?

Chronic pain is defined as pain that lasts more than twelve weeks despite medication or treatment.

How common is chronic pain?

It is the most prevalent disease worldwide, affecting millions of individuals. Chronic pain is more common in women. It is estimated that seventy percent of individuals living with chronic pain are women. However, despite impacting millions collectively, chronic pain can be an isolating experience for individuals, with many struggling to have their experiences fully understood by others.

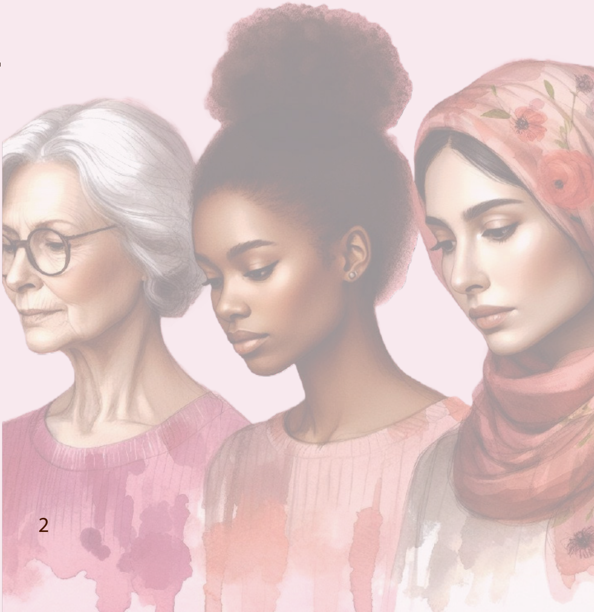

Why does pain become chronic?

Chronic pain can result from various factors. However, when there is no apparent cause, injury, or damage, chronic pain often stems from a maladaptive pain warning system, mistakenly alerting individuals to a non-existent threat. In such instances, the maladaptive warning system needs to be retrained for more accurate signalling. It is being a bit too protective and needs to learn that there is no threat or injury occurring. Pain is influenced by many factors, which will be covered in this booklet. Addressing these factors through the various self-management strategies mentioned in this booklet can contribute to reshaping the system to respond more adaptively. Almost all pain can be managed and it is important to find medical professionals who understand your concerns and recognise the importance of adopting a holistic approach to managing pain.

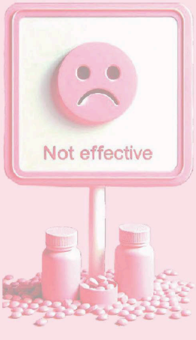

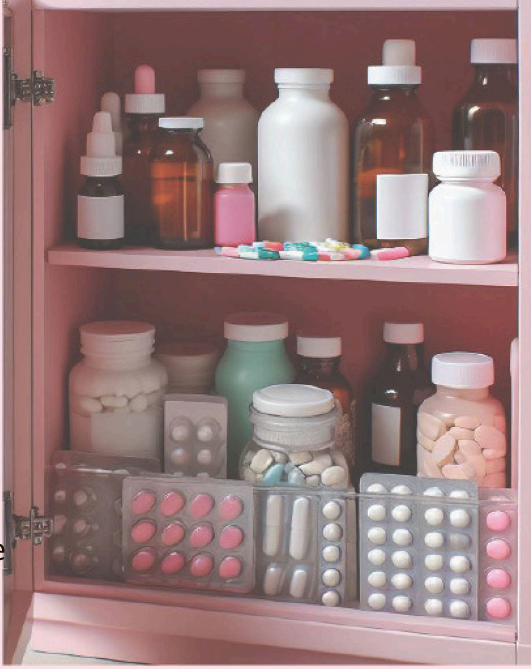

The truth about pain medication for chronic pain

While painkillers may provide temporary relief for acute (short-term) pain, their effectiveness in managing chronic pain is limited. Moreover, pain medication can have side effects, and some can be addictive. It is, however, essential to approach any changes in pain medication carefully and not abruptly stop taking them, as sudden withdrawal can lead to discomfort and other issues. Developing a carefully planned approach to reducing medication, if this is desired, with the guidance of health care providers is essential to minimising potential withdrawal symptoms.

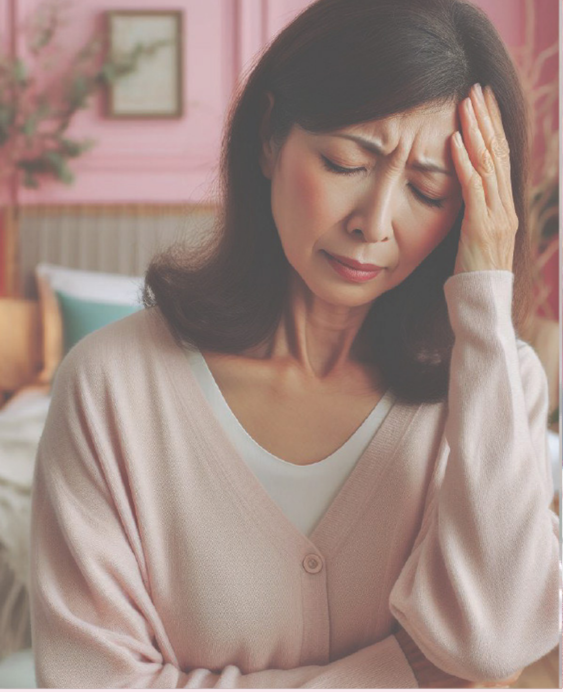

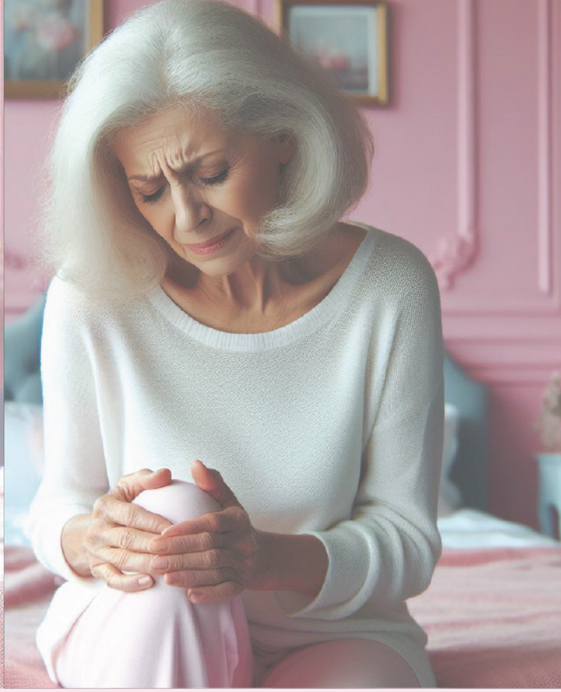

Beyond the surface: invisible but real

Chronic pain does not always come with visible wounds; there does not have to be evidence of an injury or outward signs, but its impact is profound. Unlike a cast on a broken arm or a visible injury, the absence of physical evidence may lead others to doubt or question the validity of the pain. This invisibility can contribute to scepticism and misunderstanding. The struggles and pain are real, and not acknowledging them can exacerbate the isolation and frustration experienced by those living with chronic pain.

Will I cause more damage by regular movement?

In cases of acute pain, rest may be advisable to allow the body time to heal from a specific injury or condition. However, when dealing with chronic pain, where there is no evident injury or structural damage, it is important to recognise that using the parts of the body that are painful does not mean you will be causing harm. In fact, appropriate movement and activity can be an integral part of managing chronic pain, contributing to improved function and overall well-being. Engaging in physical activity is beneficial for individuals with other chronic conditions too, but it is important to consult with your doctor to establish safe and appropriate levels of activity.

How can chronic pain affect an individual?

Chronic pain has a profound impact on various aspects of an individual's life and their well-being. Individuals may find it challenging to maintain employment, engage in certain activities, or participate in social events. The constant discomfort and the challenges of managing pain can also take a toll on individuals' mental health. Their overall quality of life can also be substantially diminished as it affects relationships, self-esteem, and the ability to enjoy life.

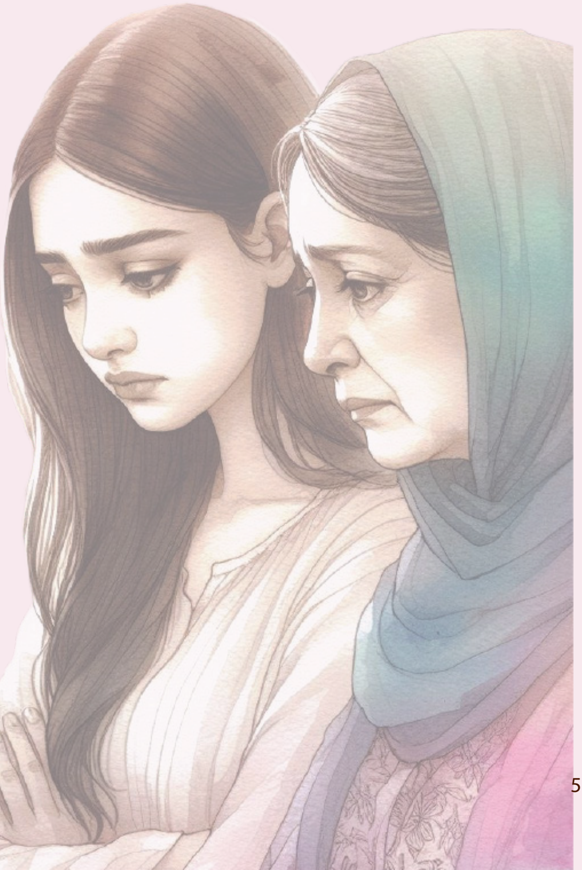

Factors that can influence pain

Biological factors such as sex, hormones, psychological factors like stress or anxiety levels, social factors including support networks, and spiritual aspects like personal beliefs can influence both the onset and intensity of pain. An individual's environment, memories, thoughts, emotions, and experiences play crucial roles in how pain is perceived and experienced. Positive emotions and strong support networks can help to mitigate pain. In contrast, negative beliefs, attitudes, and a lack of support, along with factors such as anxiety, grief, anger, and stress, can amplify pain.

Living with chronic pain alone can be a source of stress, as it can affect various aspects of life. When coupled with multiple responsibilities and duties, such as work, caregiving, or household management, the stress can intensify. Juggling these responsibilities without sufficient support or time for self-care can negatively impact health and well-being. Thus, a holistic approach is essential to managing pain effectively. Treatment strategies may include medications, physiotherapy, and lifestyle changes.

What can relatives and friends do to support patients?

The way forward involves cultivating empathy and understanding the challenges of our friends and family who may be dealing with chronic pain. Listening without judgement, offering support, and validating their feelings can make a profound difference. It is important to remember that even if we may not fully comprehend the depth of someone's pain, our willingness to empathise can contribute significantly to their well-being. Moreover, educating ourselves about the conditions and challenges our loved ones face can strengthen our ability to provide meaningful support.

The power of listening

Listening to someone in pain can provide significant relief to them, as it can feel like a substantial burden has been lifted. Often, individuals in pain experience a lack of understanding from others, and having someone truly listen and empathise can make them feel heard and supported, lightening the emotional load they may be carrying.

Acknowledge that pain is a subjective experience

It is essential to acknowledge that pain is a subjective experience, and what matters most is the individual's own description and perception of their pain, and to believe this.

Small acts of kindness

Sometimes those living with pain may need your help with shopping, cooking, or cleaning, as they often juggle these tasks all by themselves while also managing their pain. If you have time and are able to help, ask them if they need a hand. Your simple act of kindness can make a world of difference to someone in pain who is struggling.

Flexibility, patience, and an awareness of patient limitations

Chronic pain is notoriously unpredictable, and patients frequently experience fluctuations in their symptoms. Being patient and flexible with plans and activities when their pain is more difficult to manage is often required. Recognise that individuals experiencing pain may find it necessary to decline invitations or alter their activities accordingly. Prevent them from being pushed beyond their capabilities. Ensure that they do make time for activities they enjoy; it is extremely vital for their mental and physical well-being.

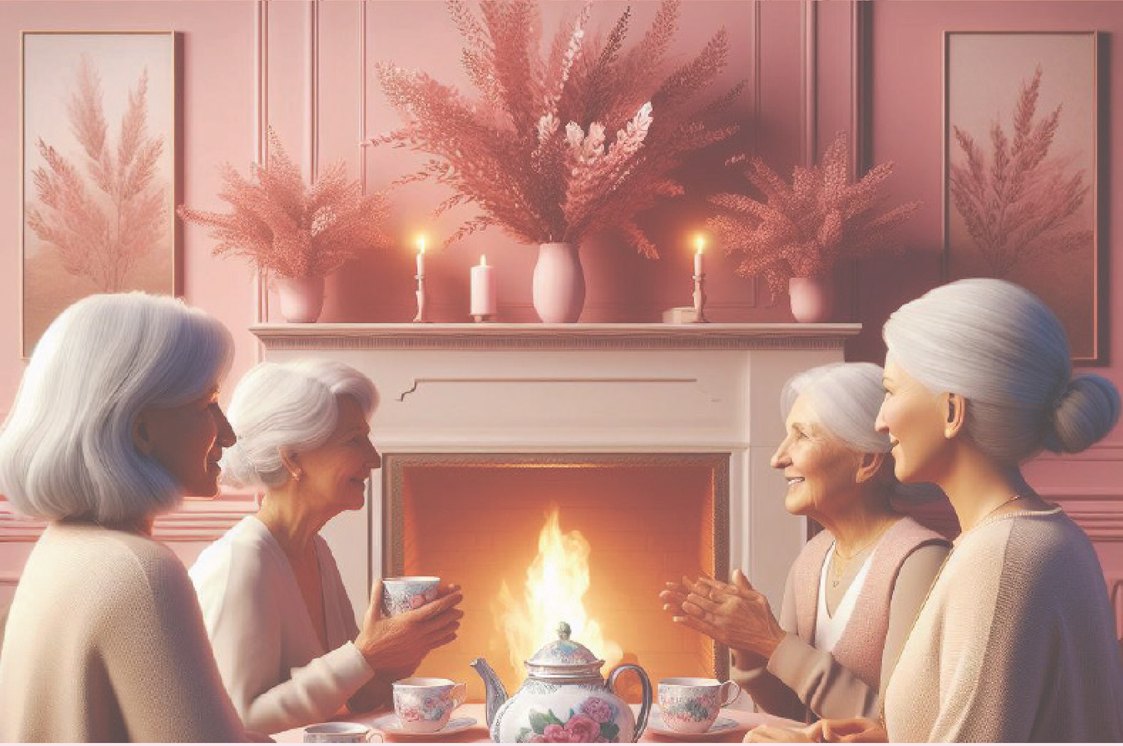

Seeking support

Patients sharing experiences

Share your experiences and challenges with someone who understands whether it is a friend, family member, or support group. Connecting with others that understand your pain can help reduce feelings of isolation. They can also allow you to discover solutions that you may not have otherwise considered.

Compassionate, candid conversations

Talking to someone who understands can provide emotional support and improve psychological wellbeing, all of which are vital in managing chronic pain.

Seeking help

It is entirely appropriate to request help and accept it. Having a strong circle of support is essential for receiving emotional and practical support during difficult times and effectively managing pain.

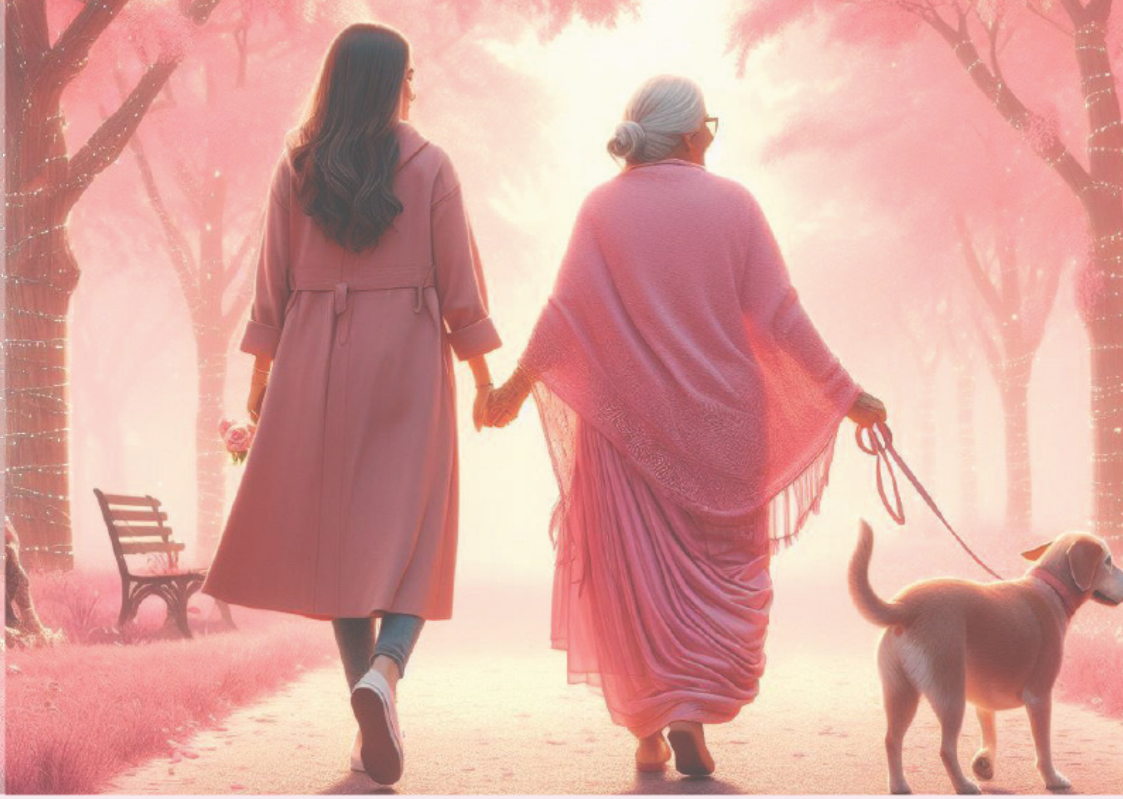

Physical activity

The positive impact of physical activity on mental health and its potential to mitigate symptoms of depression are widely recognised. Exercise also releases endorphins, which are the body’s natural painkillers. Walking is an excellent low-impact form of physical activity, is suitable for individuals of all fitness levels and requires no special equipment or skills.

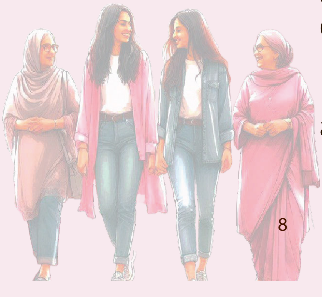

However, ensure your footwear is comfortable, non-slip, and well-fitting. Incorporating walking into one's daily routine can be an effective way to reduce stress, anxiety, and pain, improve sleep, and improve overall health and well-being. Start slowly and gradually increase the duration of your walks as you feel more comfortable. Aim to build up to thirty minutes a day, set achievable goals, and celebrate each milestone as you progress. On days when you would rather not go outside, stationary bikes provide a convenient indoor alternative.

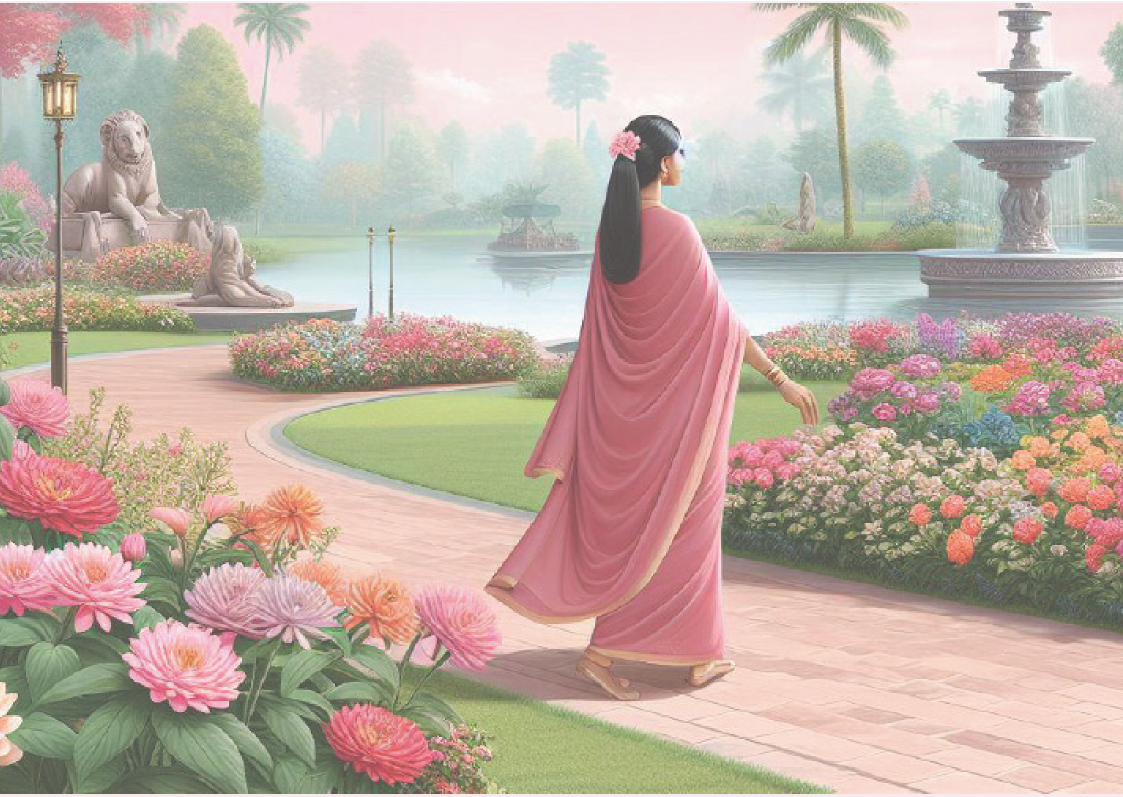

Mindfulness

There are mindfulness exercises you can attempt while you are walking. Mindfulness requires being fully absorbed and engaged in the current experience, without judgement or distraction, and having appreciation for the richness of the present moment.

Moving mindfully

Try to concentrate on the sensation of your body moving. If you go for a mindful walk, you may notice the breeze on your skin, the sensation of your feet or hands against different textures on the ground or surrounding surfaces, and the different objects, sounds and smells around you.

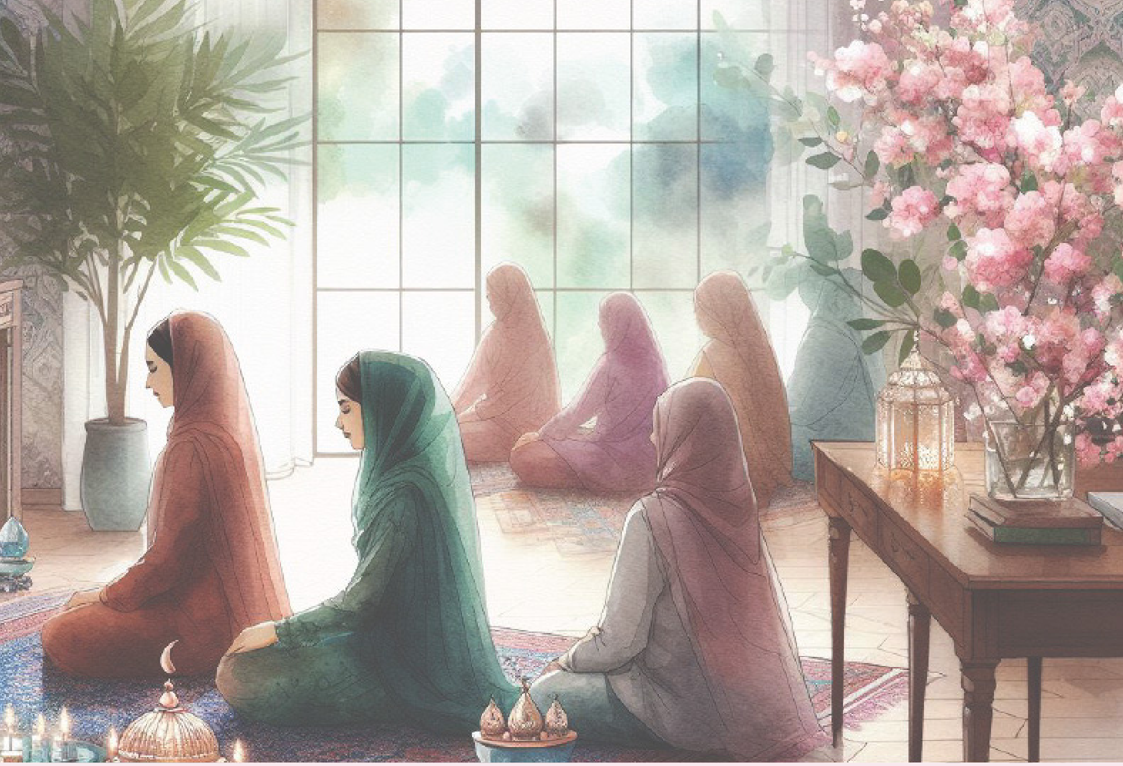

Prayer and spirituality

Prayer and spirituality can help individuals living with chronic pain. They can provide strength, purpose, hope, comfort, and inner peace and can help get individuals through challenging times. Engaging in prayer and connecting with one's spiritual beliefs often offers a source of guidance, fostering a deeper understanding of life's challenges and promoting a sense of inner peace. Taking time to explore and incorporate your own beliefs into your well-being approach can be a meaningful part of your journey in managing chronic pain.

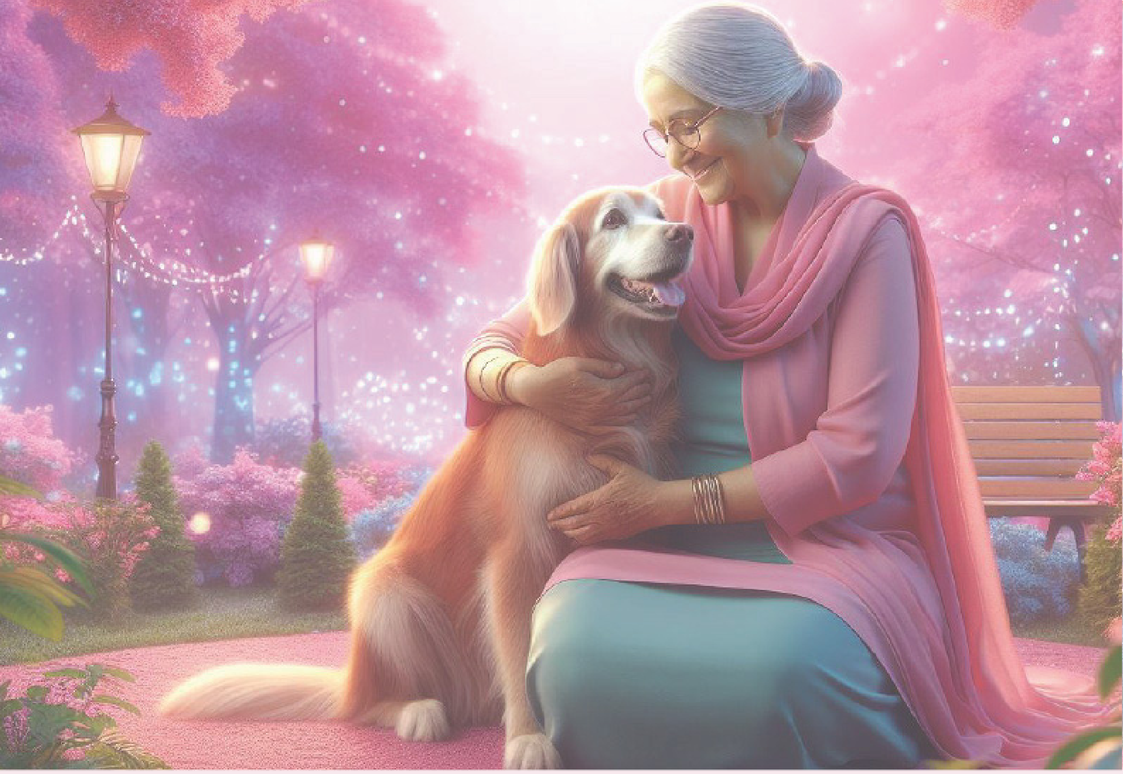

Pets can provide numerous benefits to individuals living with pain.

Some of the ways in which they have been reported to help are provided below.

Emotional support: pets can provide companionship and emotional support, which can help alleviate feelings of loneliness and depression often associated with chronic pain.

Distraction: interacting with pets can serve as a positive distraction from pain, redirecting individuals' focus and providing a sense of purpose and responsibility.

Physical activity: dogs, in particular, require regular walks and playtime, encouraging physical activity for their owners.

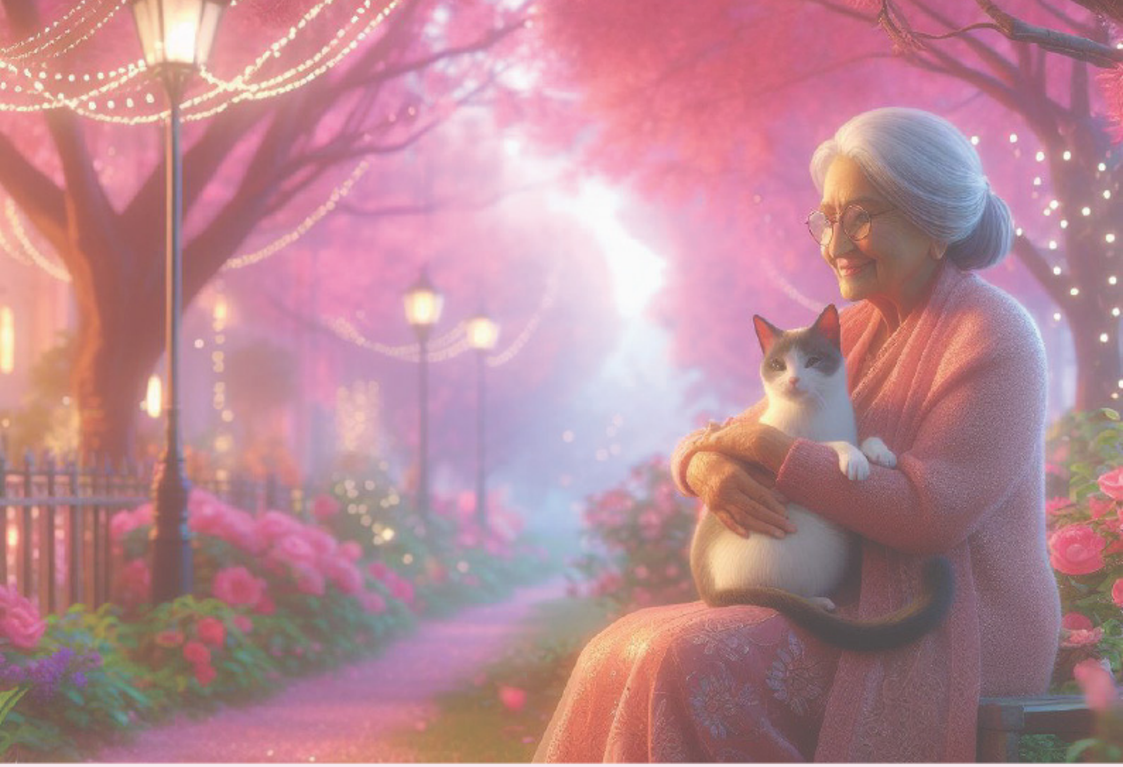

Unconditional love: pets often offer unconditional love and nonjudgmental companionship, creating a sense of comfort and security. This emotional connection can be especially valuable during challenging times.

Routine and structure: caring for a pet establishes a daily routine, providing structure and a sense of purpose. Having a predictable schedule can be beneficial for individuals managing chronic pain.

Stress reduction: interactions with pets have been shown to reduce stress levels and improve mood. This more positive emotional state can contribute to reducing pain perception.

Consideration should always be given to whether you can provide a pet with the attention, love, and care it requires before deciding to adopt one into your life.

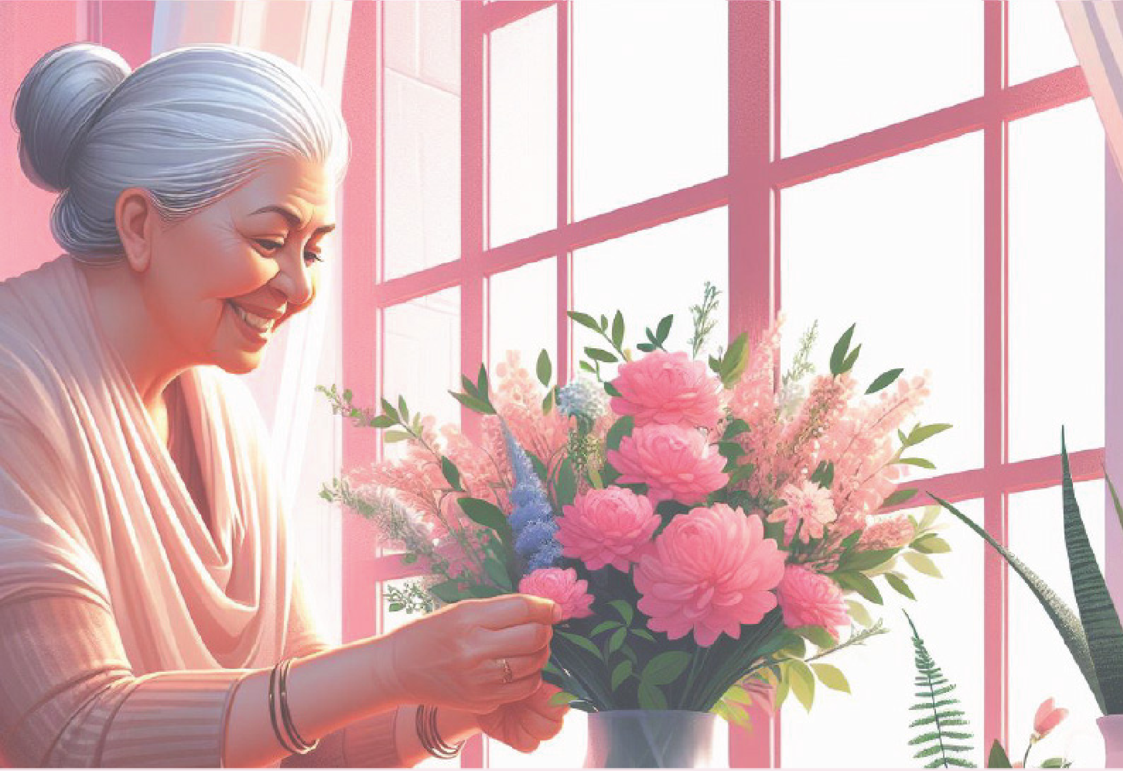

Surrounding yourself with things that bring you joy

Surrounding yourself with things that bring joy and lift your mood is a simple yet powerful way to cultivate a positive and uplifting environment. This can be a piece of art or some flowers.

Spending time with those whose company you enjoy.

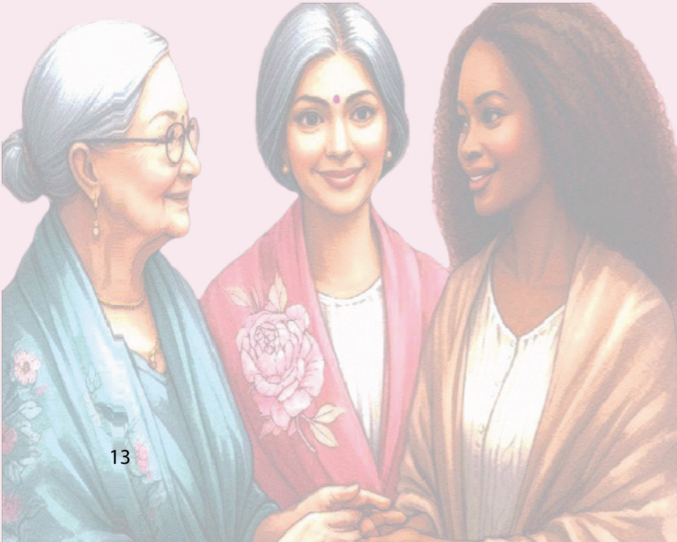

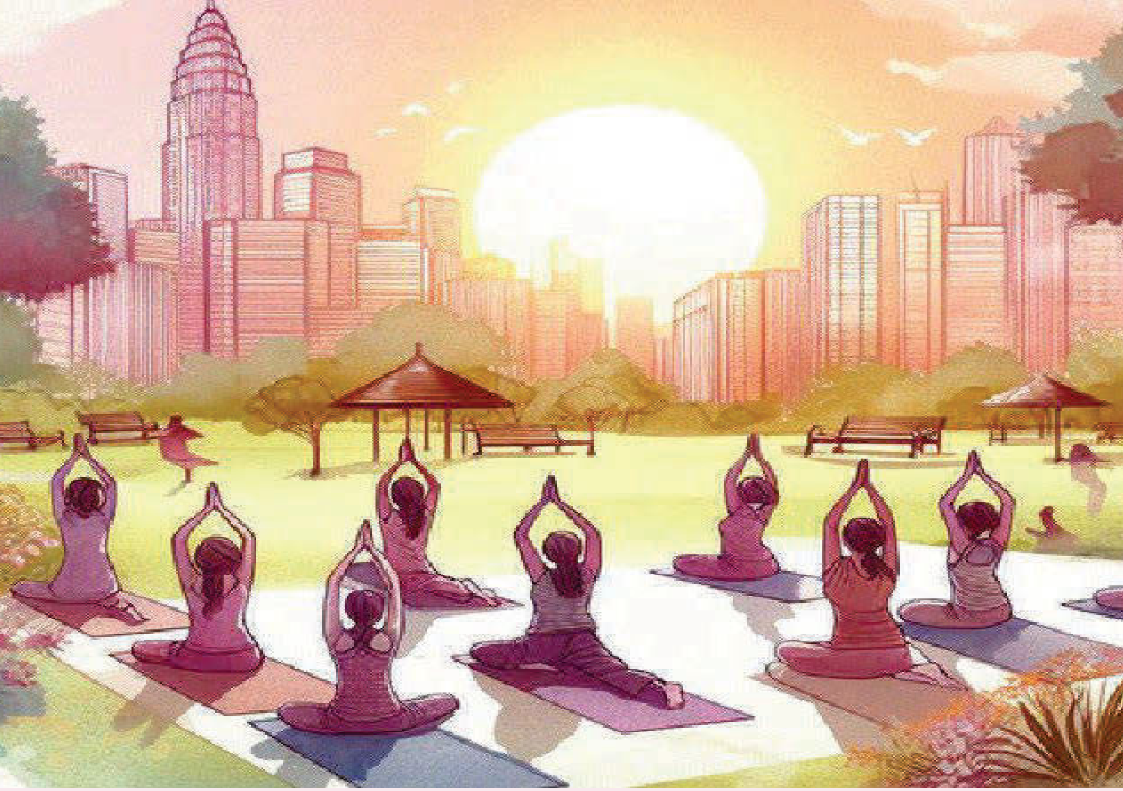

Yoga and stretching

Yoga and stretching are helpful for pain management as they help improve flexibility, increase blood flow, and reduce muscle tension and stress. Stretching can release endorphins, the body’s natural painkillers, which not only aid in pain reduction but also contribute to an improved mood. It is advisable to consult with your doctor to address any concerns about incorporating stretching into your routine. Stretching, when done regularly and with attention to your body's limits, can contribute to a more flexible and comfortable body, potentially reducing chronic pain.

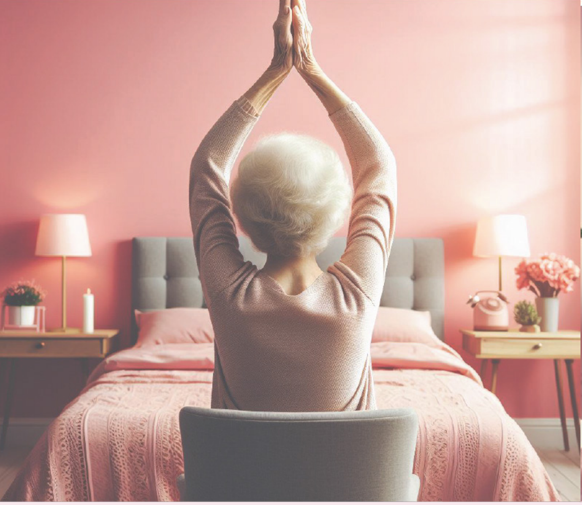
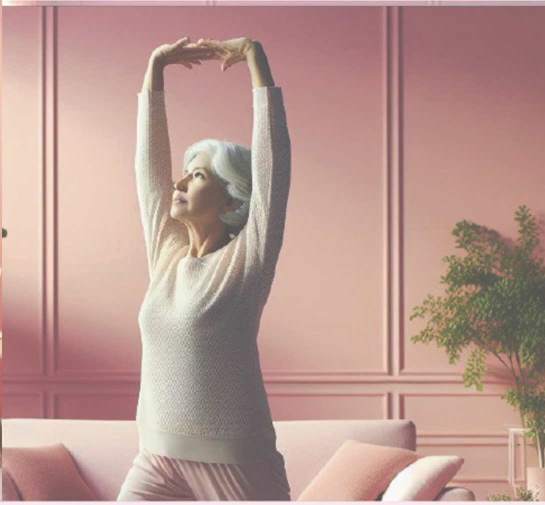

Simple Stretches

Below are some simple stretches that have been found to be helpful. Remember to move gently and within your comfort range. Feeling a mild, gentle stretch is normal and can be an indicator that you are working on flexibility. However, listen to your body, stop, and seek advice from a health care professional if you experience any sharp pain. Breathe deeply and regularly during stretches. Holding your breath may increase tension. Stretching should feel like a gradual and gentle lengthening of the muscles, not a forceful or painful experience.

Shoulders, upper back, and sides (obliques).

Sit or stand comfortably.

Clasp your hands together.

Stretch your clasped hands up towards the ceiling.

Lean gently to the right to stretch the right side; hold for a few seconds.

Return to the centre.

Lean gently to the left to stretch the left side; hold for a few seconds.
Return to the centre.

Open your arms, lowering them back down.

Repeat the entire sequence 8 to 10 times.

Shoulder Stretch

Sit or stand comfortably.

Gently roll your shoulders backward in a circular motion for 10 seconds.
Then, reverse and roll them forward for another 10 seconds.

Repeat 8 to 10 times.

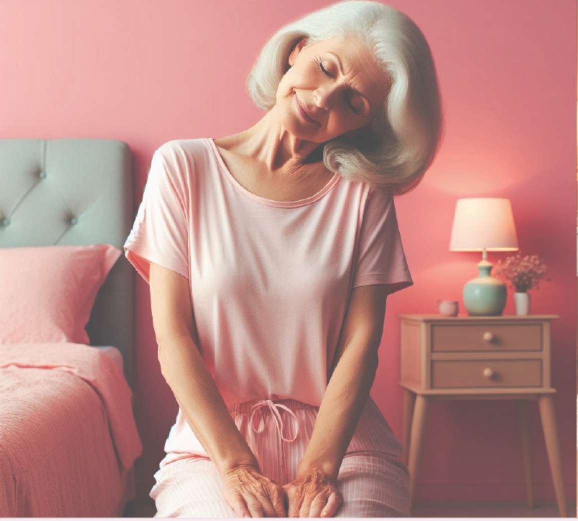

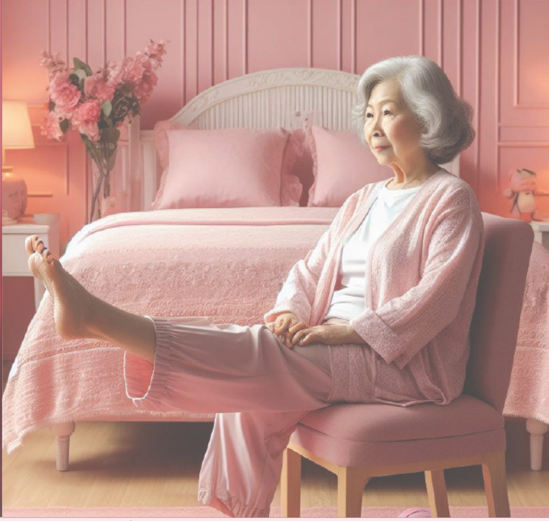

Neck Stretch

Sit or stand comfortably. Slowly tilt your head to the left side, bringing your ear toward your shoulder.

Hold for a few seconds.

Return to the centre.

Repeat on the right side.

Return to the centre.

Repeat both sides 8 to 10 times.

Back stretch

Sit on a sturdy chair with your feet flat on the floor.

Without moving your hips,turn your upper body to the left as far as is comfortable, using the back of the chair for support.

Hold the twist for a few seconds.

Return to the centre and repeat on the other side.

Repeat the sequence 8 to 10 times.

Legs and ankles:

Sit on a chair with your back straight.

Lift one leg straight out in front of you.

Point your toes outward,stretching the top of your foot and ankle.

Hold this position for a few seconds.

Then flex your foot by pulling your toes back towards you, feeling a stretch in the calf and the back of your ankle.

Hold for a few seconds and lower the leg back down.

Switch to the other leg and repeat the sequence 8 to 10 times.

Spending time in nature

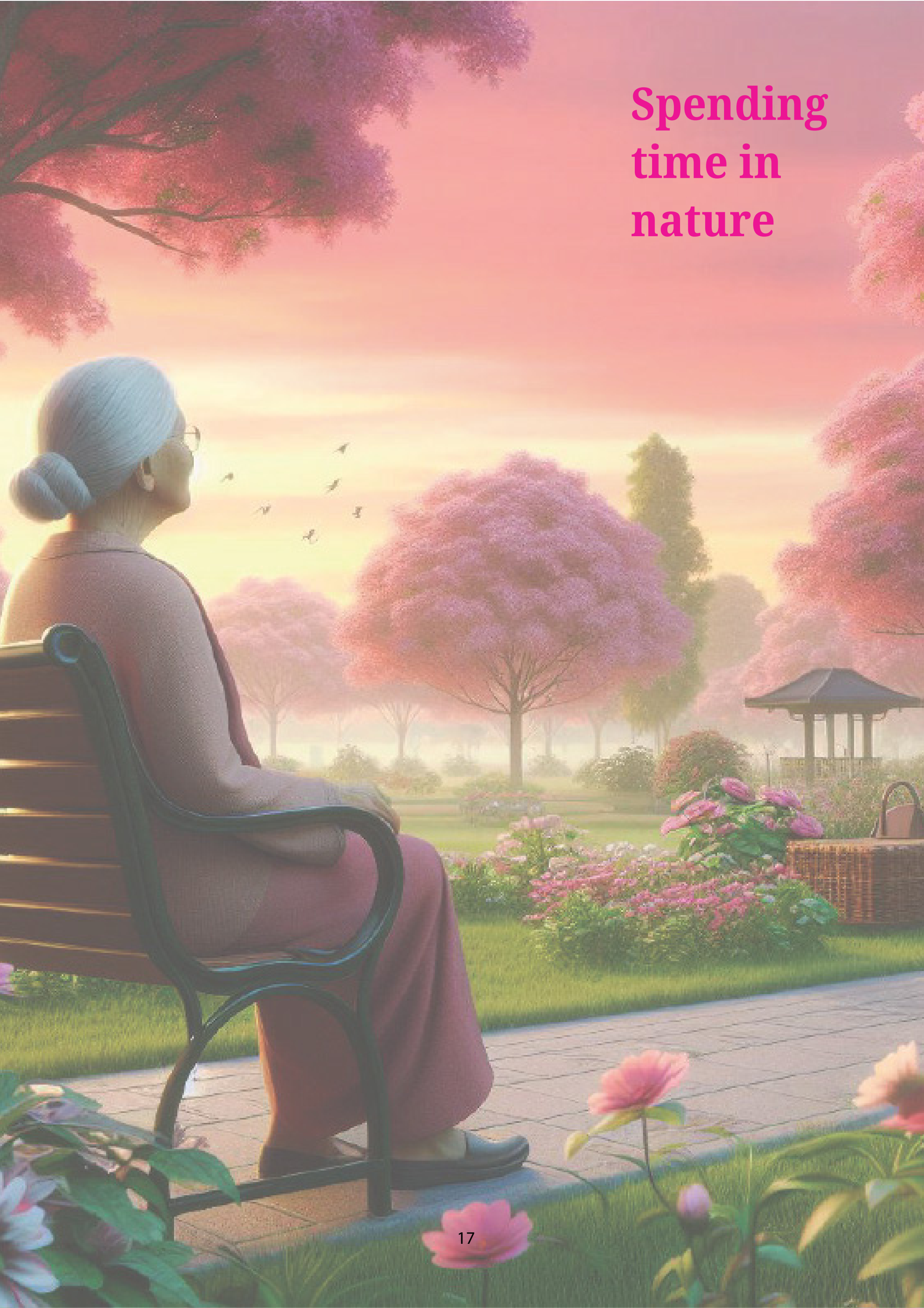

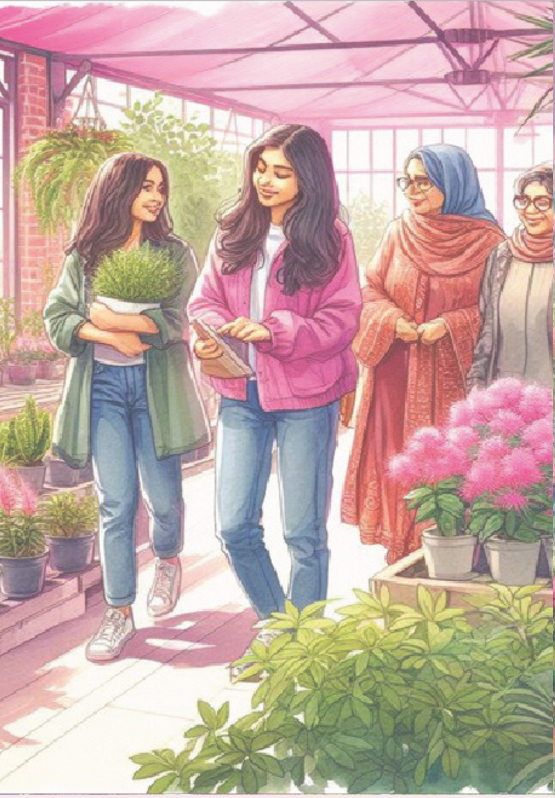

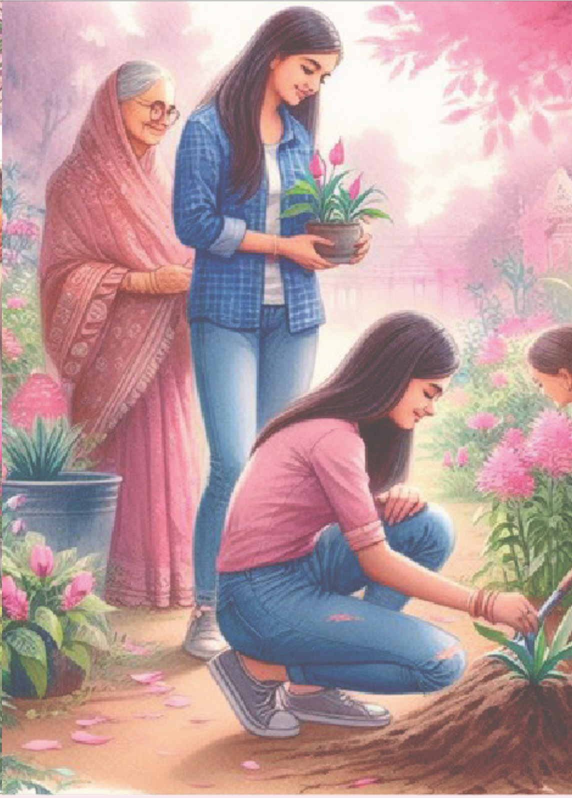

The healing power of nature

Time spent in nature has been linked to improved mood, reduced anxiety, and enhanced overall wellbeing. These can all help to reduce pain. Engaging with plants, whether through gardening or simply enjoying green spaces, encourages mindfulness and a connection with the natural world.

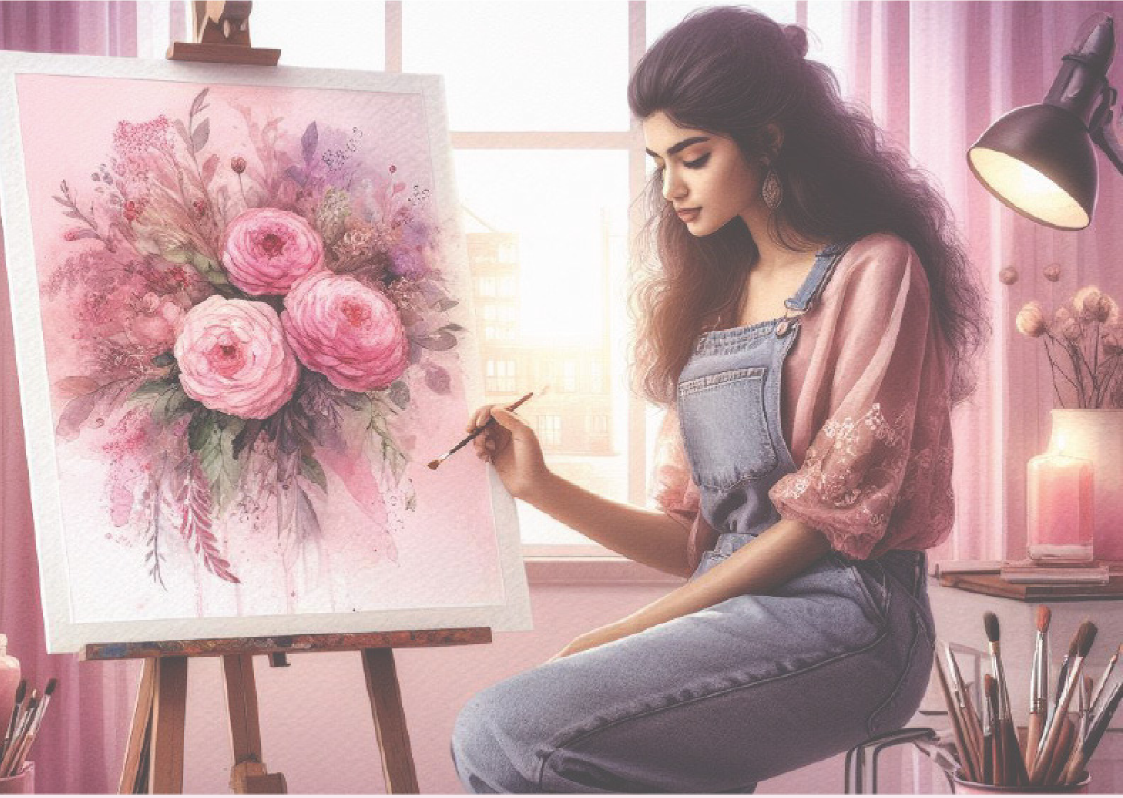

Engaging in hobbies

Investing time in hobbies and activities that bring joy and spending time with supportive individuals contribute significantly to a happier, more enjoyable, and more fulfilling life. While managing time for such activities may require careful planning, even small moments dedicated to them can have a beneficial impact. It is vital for individuals to acknowledge the importance of self-care and prioritise activities that bring them joy and relaxation, ultimately leading to improved health, well-being, and quality of life.

Crafting a path to well-being

Anxiety, depression, and feelings of isolation can be alleviated through craft. Art can also have a positive impact on one's mental health. It can provide a distraction from pain, remind individuals about their skills, and provide opportunities for socialisation. Moreover, it can also provide enjoyment, stress relief, meaning, and a sense of normality in life and enhance psychological well-being.

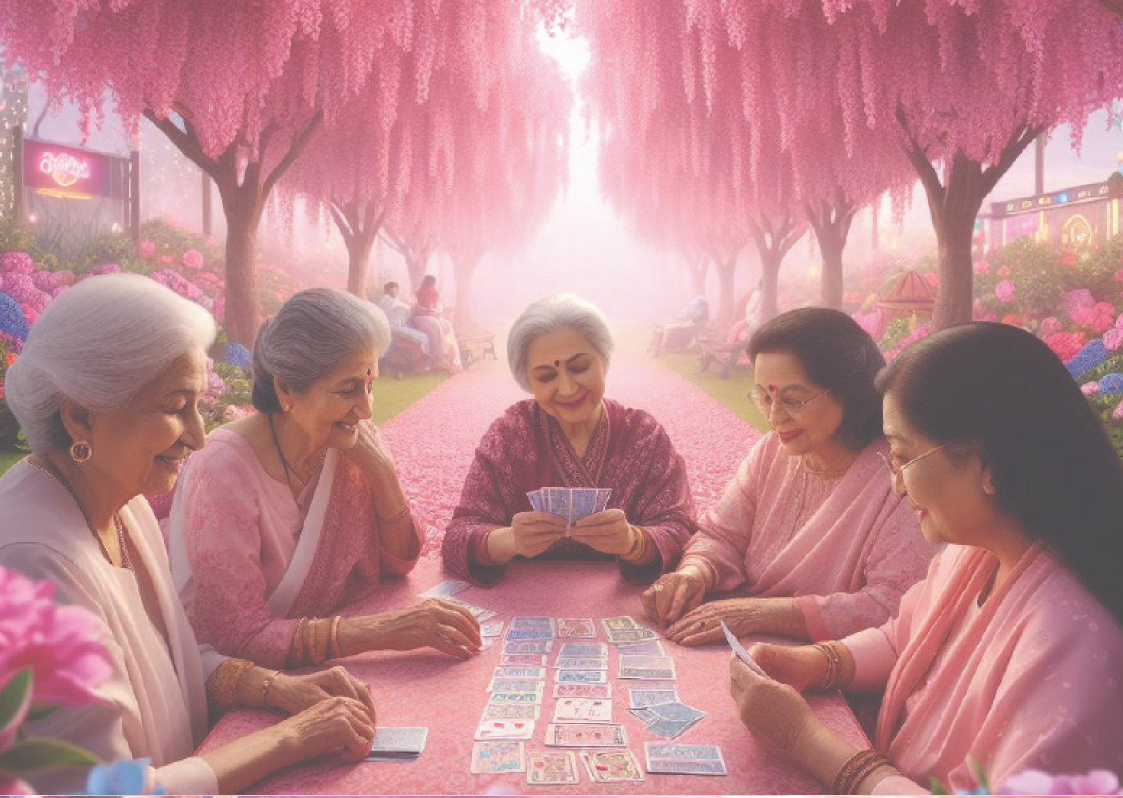

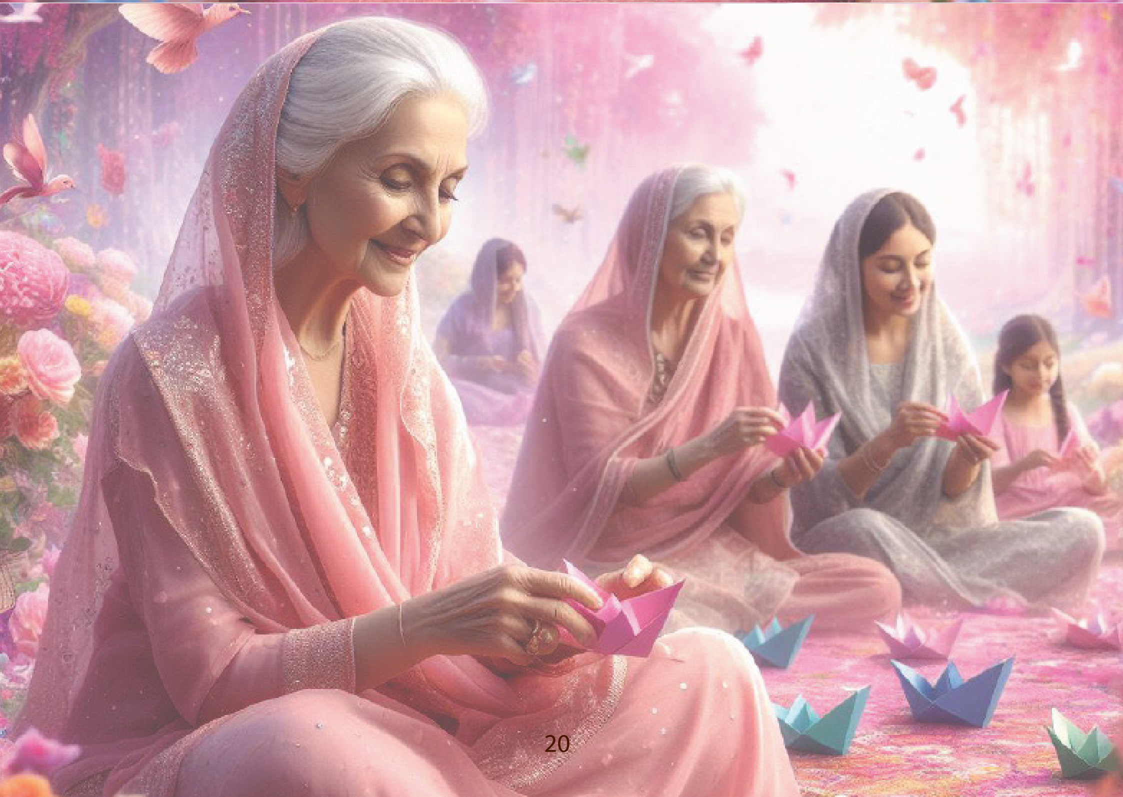

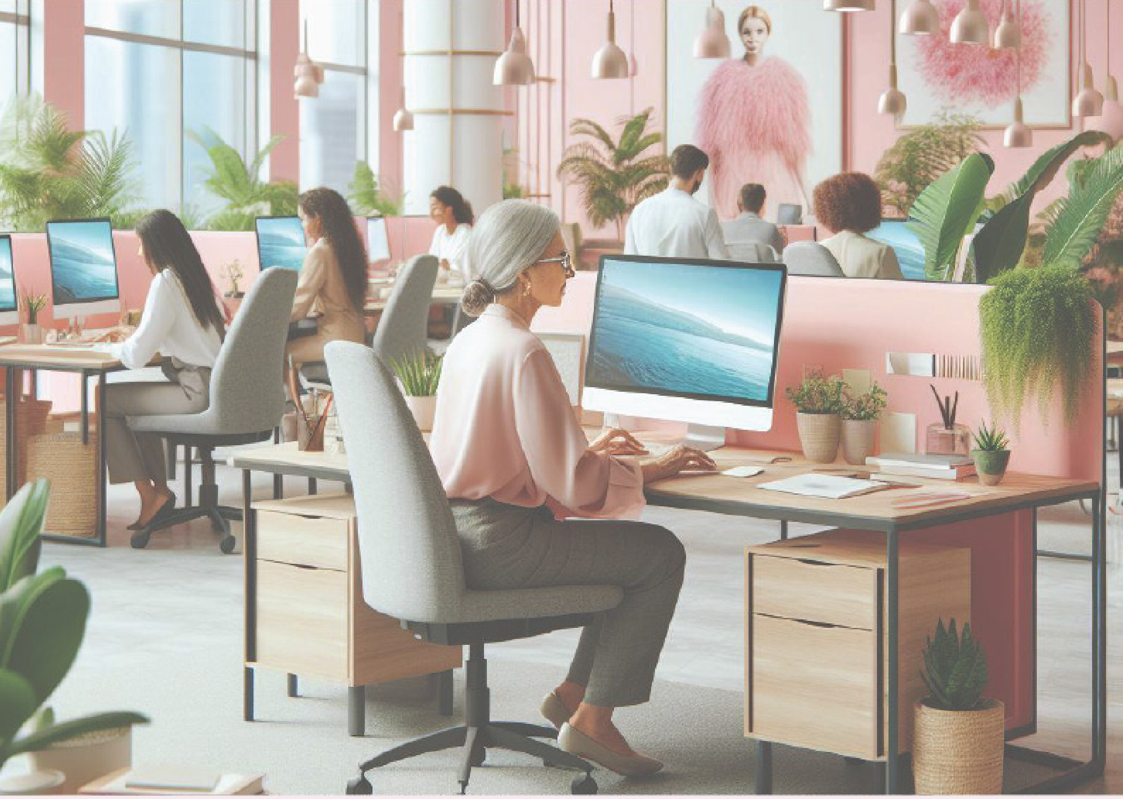

Structure, routine, and purpose

Research indicates that individuals tend to become less active and more susceptible to low moods and depression when not engaged in work. The loss of structure, routine, and purpose can impact individuals' well-being. Being at work can also serve as a distraction from pain and may not necessarily exacerbate it. If certain aspects of your job pose challenges, consider discussing these concerns with your manager.

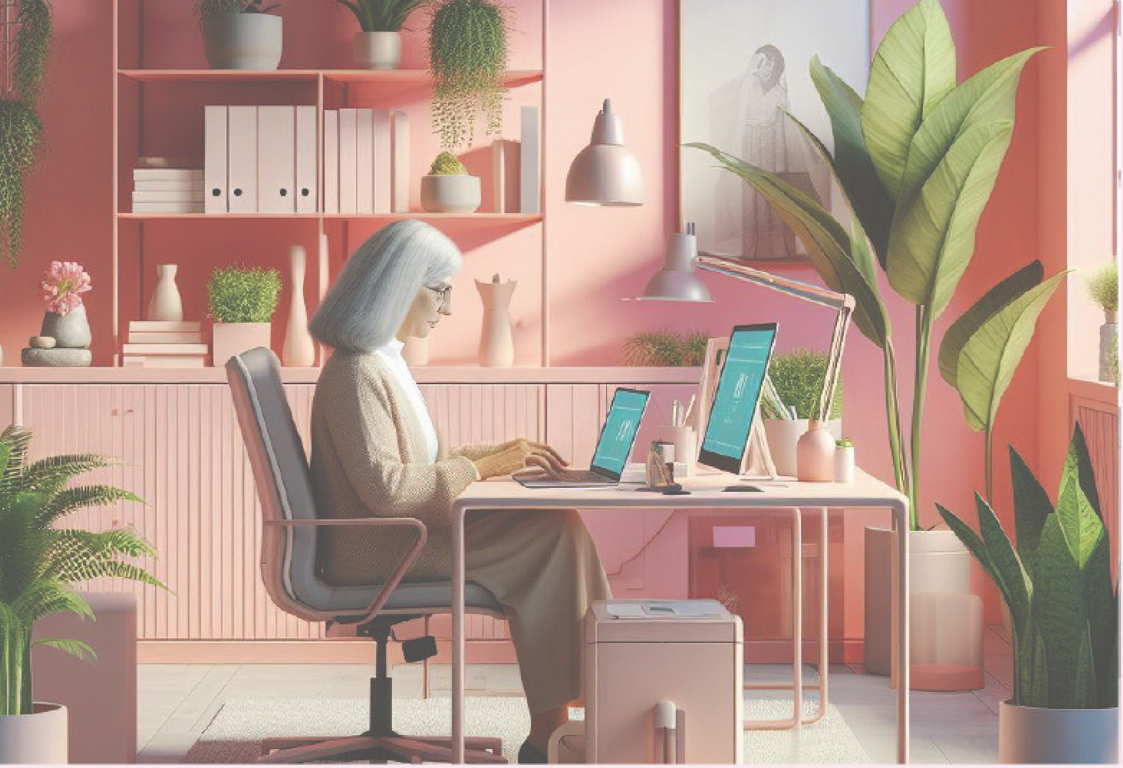

Workspace modifications, remote working, working flexible hours, and regular breaks

If you wish to continue working, emphasise your desire to do so and explore potential modifications that can make your job more manageable, or explore the possibility of working from home and working flexible hours. Open communication with your employer can contribute to a supportive work environment that aligns with your health needs. Even if working is challenging, you can still keep a daily schedule, structure, and goal by making plans to include pleasurable, beneficial, and meaningful activities.

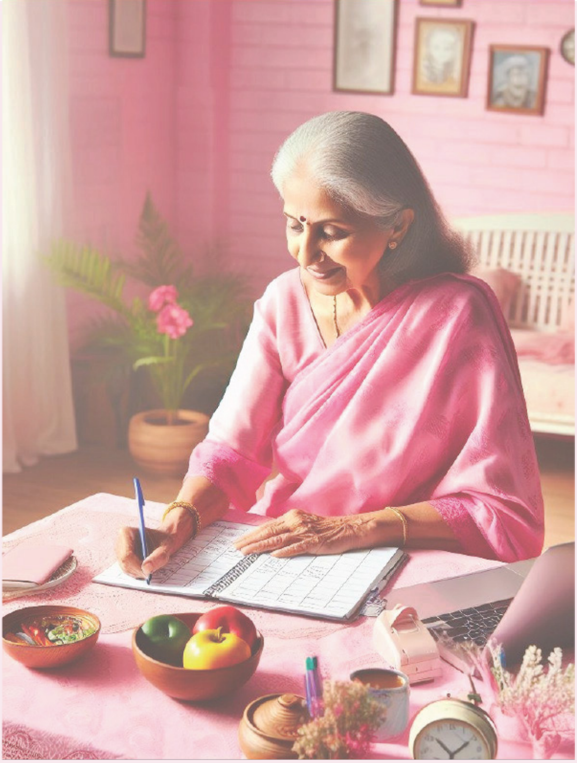

Setting SMART goals is an effective way to plan your days at home, particularly when it comes to improving physical activity. The acronym SMART stands for Specific, Measurable, Achievable, Realistic, and Timebound. An example of a simple, SMART goal is shown below.

I will walk for 15 minutes every morning before breakfast for the next three weeks, progressively increasing the time by 5 minutes each week. This will help me increase my physical activity, and at the end of three weeks, I hope to walk for 30 minutes every morning.

When faced with challenges, having a coping plan can be beneficial. Talking to someone in your support network can be one of the most effective ways to navigate difficult situations. By discussing your concerns, you can gain emotional support, different perspectives, and potential solutions. Exploring options together can provide valuable insights and contribute to a more comprehensive approach to managing whatever you may be facing.

Set goals to make time and incorporate pleasurable, beneficial, and meaningful activities.

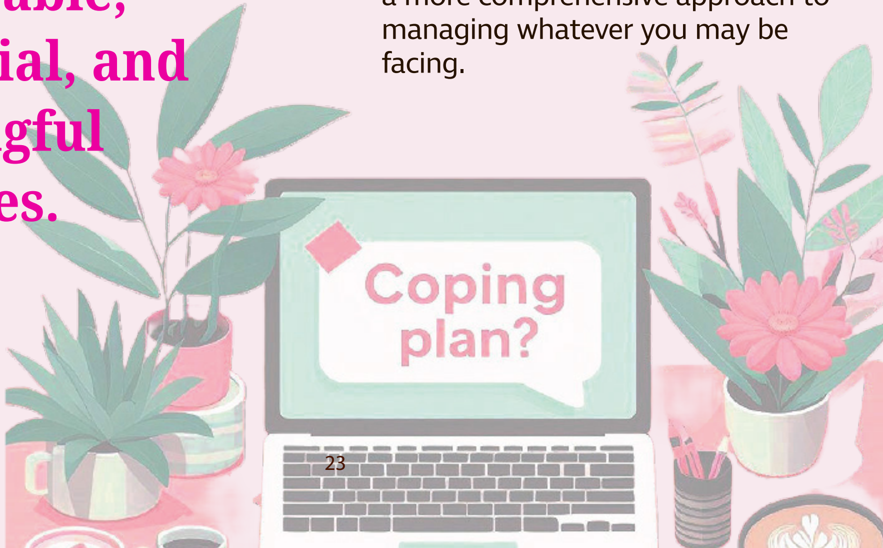

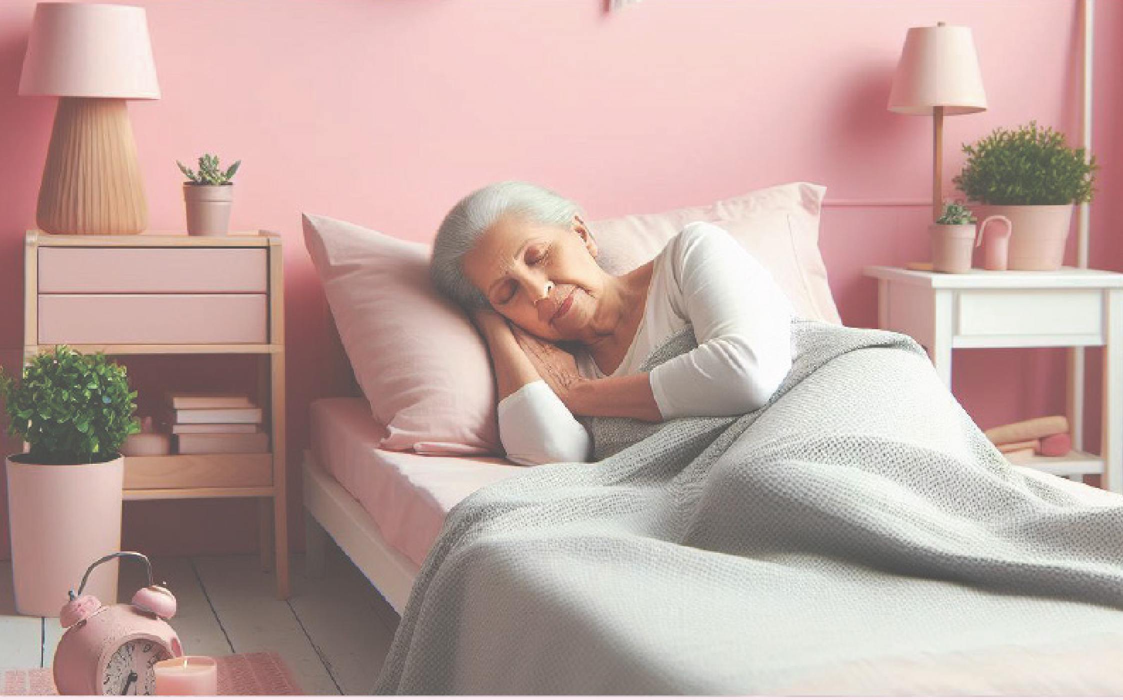

The importance of sleep

Sleep is crucial for both overall health and pain management. During sleep, the body undergoes essential processes and works towards supporting healthy brain function and maintaining your physical health and well-being. Adequate sleep is linked to improved cognitive function, emotional well-being, and the ability to cope with pain. Chronic sleep deprivation can amplify pain perception, making it more challenging for individuals to manage pain. Establishing healthy sleep patterns is integral to promoting optimal health and wellbeing, and it can significantly enhance an individual's ability to cope with pain.

Some individuals with pain have also found relief in taking a warm shower before bed, drinking a warm glass of non-dairy milk with turmeric, massaging with either ginger, clove, castor, or almond oils, and covering the massaged site with something like a knee sleeve or a glove. Additionally, avoiding caffeine and heavy meals before bedtime and maintaining a comfortable sleep environment with supportive pillows and proper bedding can help promote better sleep and minimise discomfort. A plug-in device that plays calming sounds can assist some individuals in feeling more relaxed and in improving their quality of sleep. However, it is essential for each individual to explore and adapt these methods based on their individual needs and preferences.

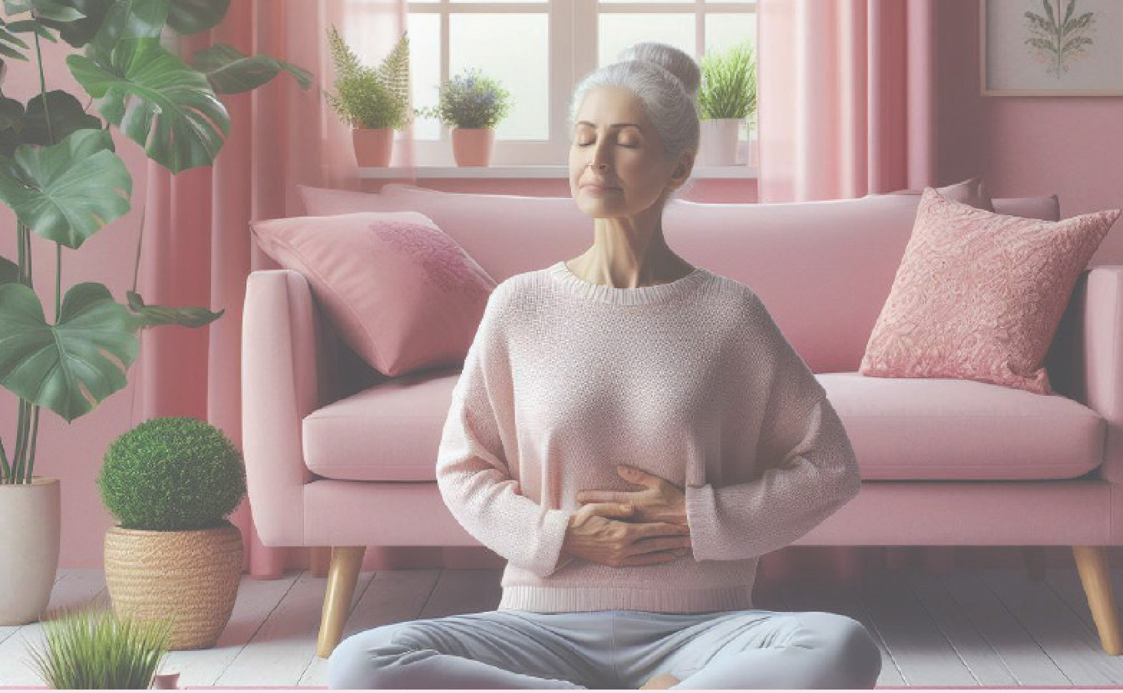

Breathwork for better sleep

Deep breathing enhances relaxation, reduces stress, and contributes to a sense of calm, which can be particularly beneficial in managing pain. Belly breathing, also known as diaphragmatic breathing, is a relaxation technique that involves deep, slow breaths to promote a sense of calm and reduce stress. Below is a simple exercise you can try for relaxation.

Sit or lie down in a comfortable position. Place one hand on your chest and the other on your abdomen, just below your ribcage. Inhale deeply (to a count of four or what feels comfortable for you) through your nose, allowing your abdomen to expand, feeling your hand on your abdomen rise while keeping your chest relatively still. Exhale slowly through your mouth or nose, allowing your abdomen to fall (to a count of six or whatever feels comfortable to you, but it should be longer than the inhale).

Focus on a slow and controlled exhalation, feeling your hand on your abdomen lower. Adjust the counts to what feels comfortable for you but ensure that the exhale is longer than the inhale. Continue this slow, rhythmic breathing pattern for a minute and work up to five to ten minutes per session. Incorporating belly breathing into your routine helps promote a sense of calm and reduce muscle tension.

Other tips

Batch cooking and freezing minimises the effort required for daily cooking, reducing stress and fatigue. Multi-cookers can be helpful.
These appliances simplify meal preparation, requiring less hands-on time and effort, making cooking a more manageable task.
Certain herbs and spices possess anti-inflammatory properties that may provide relief from pain and contribute to overall well-being. It is crucial to ensure that they do not interact with any medications. You can try and create herbal teas using ingredients like turmeric, cloves, or ginger. Flaxseeds may be helpful in reducing inflammation due to their high content of omega-3 fatty acids, which have been associated with anti-inflammatory effects.
Hydration supports overall health, including joint and muscle function, contributing to pain management.
Applying heat (warm towels, heated massage devices), commonly used to relax and soothe muscles, stimulate blood flow, and relieve stiffness, or cold (cold packs), typically used to reduce inflammation and numb pain, can be beneficial.

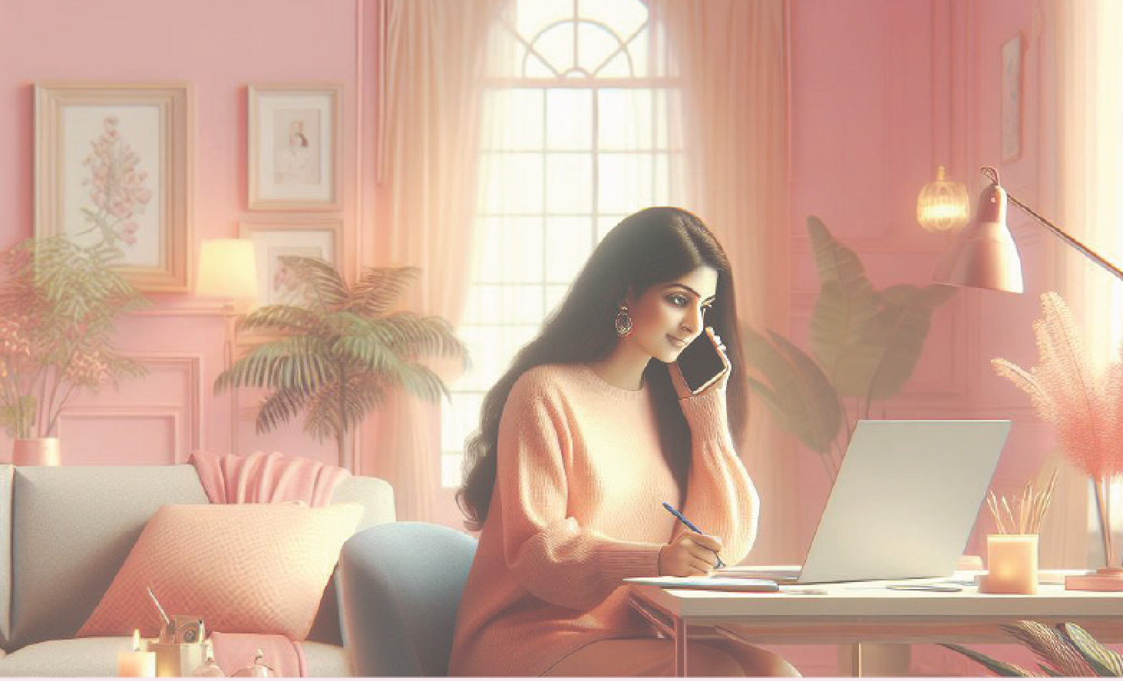

Further information

More information can be obtained from the websites below.

https://healthtalk.org/chronic-pain/learning-about-pain-management

Healthtalk.org provides information and resources on various health conditions, including chronic pain. Many individuals have shared their stories, aiming to provide valuable insights and support to others facing similar challenges.

Pain UK is a charity that also has information that you may find helpful.

https://painuk.org/help-and-support/

The Mental Health Foundation and Mind both provide support and resources on mental health and well-being.

https://www.mentalhealth.org.uk
https://www.mind.org.uk/

Headspace: a website for mindfulness

www.headspace.com

For those interested in exploring cognitive Behavioural Therapy: www.getselfhelp.co.uk

Any sudden, severe pain requires medical attention.

References

Biegler, P. (2023). *Why does it still hurt? How the power of knowledge can overcome chronic pain.* Scribe.

Carr, E. C. J., Norris, J. M., Hayden, K. A., Pater, R., & Wallace, J. E. (2020). A Scoping Review of the Health and Social Benefits of Dog Ownership for People Who Have Chronic Pain. *Anthrozoös, 33*(2), 207-224.

Centers for Disease Control & Prevention. (2019). *About pets and people.* https://www.cdc.gov/healthypets/health-benefits/index.html

Harvard Health Publishing. (2020). *Exercising to Relax.* https://www.health.harvard.edu/staying-healthy/exercising-to-relax

Live well with pain. (2024). What is Self-management. https://livewellwithpain.co.uk/what-is-self-management/

Mind (2024). *Mindfulness.* https://www.mind.org.uk/information-support/drugs-and-treatments/mindfulness/mindfulness-exercises-tips/

NHS (2021). *Ways to manage chronic pain.* https://www.nhs.uk/live-well/pain/ways-to-manage-chronic-pain/

NHS England and NHS Improvement South West. (2020). *Opioid prescribing for chronic pain.* https://www.england.nhs.uk/south/info-professional/safe-use-of-controlled-drugs/opioids

NHS Inform. (2018). *Chronic Pain.* https://www.nhsinform.scot/illnesses-and-conditions/brain-nerves-and-spinal-cord/chronicpain#:~:text=Chronic%20or%20persistent%20pain%20is,of%20an%20injury

Acknowledgments

The authors would like to thank all the women who generously gave up their time and shared their experiences and advice, and appreciate their substantial contributions to the production of this resource.

Image credits

Images generated with the assistance of AI

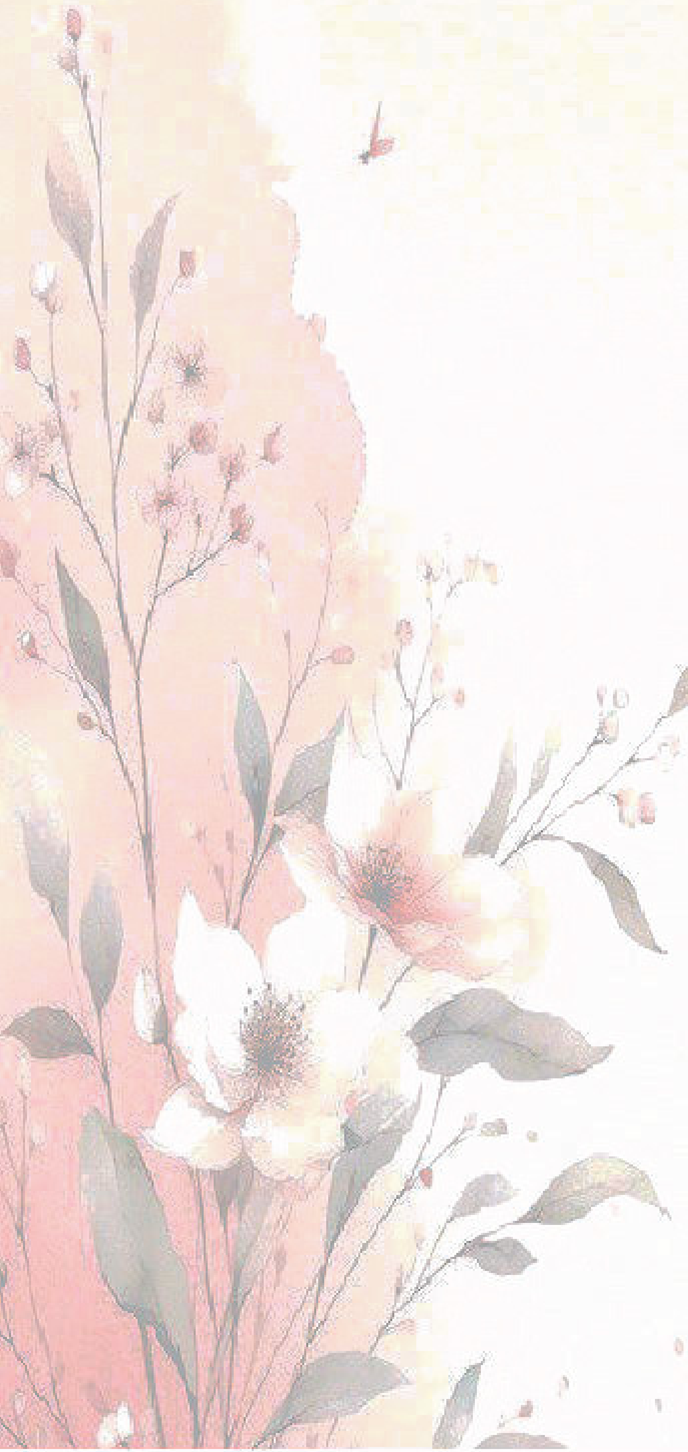

Biring, S., Burton, A., &
Kevern, P.

STAFFORDSHIRE UNIVERSITY

School of Health,
Science, and
Well-Being 2024
